# YBX1 Modulated Corneal Neovascularization Induced by Alkali Burn via m^5^C-Dependent Regulation of the STAT3/HIF-1α/VEGFA Axis

**DOI:** 10.1167/iovs.67.2.42

**Published:** 2026-02-23

**Authors:** Zixian Yang, Yulin Yan, Qian Deng, Yuelan Gao, Jiewen Mao, Lan Ke, Shanshan Wan, Yanning Yang

**Affiliations:** 1 Department of Ophthalmology, Renmin Hospital of Wuhan University, Wuhan, People's Republic of China

**Keywords:** corneal neovascularization (CoNV), alkali burn (AB), angiogenesis, 5-Methylcytosine (m^5^C), Y-box binding protein 1, STAT3, HIF-1α, VEGFA, apoptosis

## Abstract

**Purpose:**

Effective management of corneal neovascularization (CoNV) remains challenging, and the role of epitranscriptomic regulation, particularly N5-methylcytosine (m^5^C) modification, in this process remain incompletely defined. This study investigated the function and mechanism of the RNA-binding protein YBX1 in CoNV following alkali burn (AB).

**Methods:**

An AB-induced CoNV model was generated using C57BL/6 mice. In vitro, human umbilical vein endothelial cells (HUVECs) underwent hypoxia/reoxygenation (H/R). Multi-omics approaches including transcriptome sequencing, RNA immunoprecipitation sequencing, and m^5^C methylated RNA immunoprecipitation sequencing were used to identify YBX1 targets and their modification status. Functional assays assessed angiogenesis, apoptosis, and reactive oxygen species (ROS). The therapeutic potential of the YBX1 inhibitor Soyasaponin II (SII) was evaluated in vivo.

**Results:**

YBX1 was upregulated following AB and H/R. YBX1 knockdown suppressed HUVEC migration, tube formation, and ROS production, while promoting apoptosis; these effects were rescued by HIF-1α overexpression. Mechanistically, YBX1 activated the JAK1/STAT3 pathway and recognizes m^5^C-modified sequences on STAT3 and VEGFA mRNAs, enhancing their stability. In vivo, subconjunctival injection of SII attenuated CoNV, reduced inflammation, and modulated macrophage polarization.

**Conclusions:**

Our study unveils a novel epitranscriptomic mechanism in which YBX1 drives CoNV by regulating the stability of m^5^C-modified STAT3 and VEGFA mRNAs, thereby activating the JAK1/STAT3/HIF-1α axis. Inhibition of YBX1 with SII effectively counteracts this pathway, highlighting YBX1 as an attractive candidate for intervention against sight-threatening CoNV.

The cornea, together with the tear film, provides approximately 70% of the eye's refractive power, making it a critical component of the ocular optical system. Its avascular nature is indispensable for maintaining transparency and optimal visual function.^1^^,^^2^ Alkali burn is a particularly severe form of ocular injury, commonly leading to corneal inflammation, stromal edema, and neovascularization, ultimately leading to profound visual impairment or blindness.^3^ Corneal neovascularization (CoNV) presents a significant global burden, with approximately 1.4 million new cases annually, yet treatment options are inadequate.^4^ Standard anti-inflammatory treatment with corticosteroids is limited by side effects like glaucoma, cataract formation, and heightened susceptibility to ocular infections.^5^ Corneal transplantation can restore transparency in severe cases; however, its application is hindered by factors such as high financial burden, immune rejection, and inconsistent patient adherence.^6^^,^^7^ The pathophysiological role of VEGFA in ocular angiogenesis is well-established. It is secreted by various cell types, including corneal cells, in response to inflammation or injury.^8^ Although anti-VEGFA therapy has shown efficacy in retinal neovascular disorders, its therapeutic benefit on the ocular surface remains limited, with inconsistent outcomes in clinical practice.^4^^,^^9^ Thus, the development of effective interventions for CoNV remains challenging. Therefore, identifying new molecular targets is imperative for developing more effective pharmacological strategies to treat this vision-threatening condition.

Characterized by a conserved cold shock domain (CSD) that binds both DNA and RNA, Y-box binding protein 1 (YBX1) is a multifunctional regulator of gene expression at transcriptional and post-transcriptional levels.^10^ The multifunctional nature of YBX1 enables it to critically modulate gene expression and engage in numerous cellular activities.^11^ Extensive studies have demonstrated that YBX1 contributes to cellular adaptive responses under stress conditions, maintains genomic stability, regulates mRNA stability and translation, and collectively influencing cell fate.^12^ Dysregulated YBX1 is linked to various pathological conditions, including disorders marked by excessive proliferation, invasion, tumorigenesis, drug insensitivity, inflammatory responses, and aberrant angiogenesis.^13^^–^^15^ Recent evidence suggests that targeting YBX1 through antisense oligonucleotides (ASOs) can suppress pro-angiogenic signaling pathways, including Bcl-xL/VEGFR2 and Bcl-xL/Tie, thereby inducing apoptosis in vascular endothelial cells.^16^ El Bakkouri et al. further reported that YBX1 may contribute to the abnormal proliferation of endothelial cells during retinal vascular development in murine.^17^ Additionally, Zhang et al. proposed that YBX1 may function as an RNA-binding protein (RBP) involved in pathological ocular angiogenesis.^18^ Consequently, we developed an interest in whether YBX1 is involved in CoNV, and there is an urgent need to explore its precise regulatory mechanisms.

N5-methylcytosine (m^5^C) is a pervasive epigenetic modification found in both DNA and various classes of RNA. YBX1 modulates various phases of mRNA metabolism, including pre-mRNA splicing, nuclear export, stability, degradation, and translation.^19^ The NOP2/Sun RNA methyltransferase (NSUN) family has been identified as key m^5^C writers of diverse RNA. The most extensively studied member, NSUN2, serves as a principal m^5^C methyltransferase with roles in cell cycle regulation and tumorigenesis. On the other hand, the ALY/REF export factor (ALYREF) serves as a canonical m^5^C reader, capable of recognizing and binding m^5^C-modified mRNA transcripts in both the nuclear and cytoplasmic compartments.^20^^,^^21^ In addition to ALYREF, YBX1 has recently emerged as another m^5^C reader. As a multifunctional nucleic acid-binding protein, YBX1 identifies m^5^C sites on mRNA and participates in post-transcriptional regulation of mRNA fate.^22^

Tissue hypoxia is recognized as a critical pathogenic factor in corneal injury, predominantly mediated by hypoxia-inducible factor-1 (HIF-1).^23^ HIF-1 functions as a heterodimeric transcription factor consisting of HIF-1α and HIF-1β subunits, which collectively regulate a wide range of hypoxia-responsive genes. Under normoxia, HIF-1α undergoes rapid oxygen-dependent degradation. Hypoxic stress, however, stabilizes HIF-1α, allowing it to enter the nucleus, dimerize with HIF-1β, and bind to hypoxia-response elements (HREs) in target gene promoters. This initiates transcriptional programs involved in angiogenesis, apoptosis, and inflammasome activation.^24^ Emerging evidence suggests that YBX1 facilitates HIF-1α translation by binding to and resolving complex stem-loop structures in its 5ʹ UTR region.^25^ Hypoxia promotes the proliferation of vascular endothelial cells, whereas apoptosis induction can suppress CoNV.^26^ Bcl-2 is a classical anti-apoptotic protein, whereas its counterpart Bax promotes apoptosis.^27^ The Janus kinase/signal transducer and activator of transcription (JAK/STAT) signaling cascade is central to hypoxic signaling. During hypoxia, HIF-1α associates with STAT3, resulting in the transcriptional upregulation of target genes. Notably, pharmacological inhibition of JAK1/STAT3 phosphorylation markedly reduces cell viability and downregulate genes related to angiogenesis, tumor metastasis, and proliferation.^28^ Additionally, prior studies have demonstrated that alkali burn (AB) activate the NLRP3–ASC–caspase-1–IL-1β pyroptotic pathway in cornea epithelial cells.^29^ The role of YBX1 in the HIF-1α/JAK1/STAT3 pathway during CoNV, however, remains unexplored.

We therefore propose that YBX1 may regulate AB-induced CoNV through multiple pathways. This study aims to elucidate these mechanisms and identify novel therapeutic targets for CoNV management.

## Materials and Methods

### Antibodies and Reagents

MedChemExpress (Monmouth Junction, NJ, USA) supplied both the YBX1 inhibitor Soyasaponin II (SII) and Actinomycin D, dissolved in dimethyl sulfoxide (DMSO). All drug solutions are ensured to contain a final concentration of 1% DMSO to minimize its cytotoxicity and potential impact on corneal healing.^30^^,^^31^ All siRNA oligonucleotides and polymerase chain reaction (PCR) primers were designed and supplied by Sangon Biotech (Shanghai, China). The sequences of siRNAs are available in Supplementary Table S1. Overexpression plasmids were obtained from Miaoling Bio (Wuhan, China), and lentiviral particles were supplied by HanBio Technology Co., Ltd. (Shanghai, China). Plasmid map diagrams are shown in Supplementary Figure S1. Primary antibodies were acquired from the following commercial sources: anti-YBX1, anti-VEGFA, anti-β-actin, anti-NSUN2, and anti-ALYREF were purchased from Proteintech (Wuhan, China); anti-HIF-1α, anti-phospho-JAK1 (Tyr1022, p-JAK1), and anti-JAK1 from Affinity Biosciences (Jiangsu, China); anti-CD31 from R&D Systems (Minneapolis, MN, USA); anti-F4/80, anti-CD86，anti-Cleaved PARP, anti-Cleaved Caspase-3, anti-phospho-STAT3 (Tyr705, p-STAT3), and anti-STAT3 from Cell Signaling Technology (Danvers, MA, USA); anti-NLRP3 from Abclonal (Wuhan, China); anti-Bcl-2 and anti-Bax from HUABIO (Hangzhou, China); anti-CD163 from Abcam (Danaher, USA). Additional experimental details, including catalog numbers and working dilutions, are provided in Supplementary Table S2.

### Experimental Animals and CoNV Model

All experiments used male C57BL/6 mice (6–8 weeks, 20–25*g*, SPF grade) sourced from Wuhan University. The protocol was approved by the local animal-ethics committee (Approval No. 20220504A). Animals were housed under standard conditions (20–22°C, 12 hours of light/dark) with free access to food and water. To create the AB model, mice were anesthetized with pentobarbital sodium (50 mg/kg intraperitoneal injection [IP]), and a disc soaked in 1 M NaOH (2.5 mm diameter) was placed on the cornea for 25 seconds. Residual alkali was removed by saline irrigation.^32^ Post-injury treatment with DMSO or SII (subconjunctival injection) was administered every 2 days from day 1 to day 14. At the endpoint, corneas were dissected on ice after euthanasia.

Randomization produced the following groups: a control group (*n* = 18 each) and AB groups at post‑injury days 3, 5, 7, and 14 (*n* = 18 each) to define CoNV dynamics. To determine the optimal SII concentration, groups included control, AB, AB + DMSO, and AB + SII (1, 3, 5 mg/kg, *n* = 18 each). Three independent replicates were conducted for every assay.

### Corneal Opacity and Neovascularization Evaluated by Slit-Lamp Microscopy

Neovascularization and opacity were assessed by slit-lamp biomicroscopy at 5, 7, and 14 days following AB. The area of corneal neovascularization (A) was quantified using the following formula:

$$\begin{array}{c} A=C/12\;\times \;3.14\;\times \;\left[r^{2}-\;{\left(r-l\right)}^{2}\right] \end{array}$$

where *C* represents the number of clock hours during which new blood vessels cover the limbus, *r* represents the radius of the mouse cornea, and *l* represents the length of new blood vessels from the limbus. Corneal opacity was scored based on the following standardized grading system^33^: 0 = completely transparent; 1 = mild cloudiness; 2 = moderate opacity; 3 = severe opacity with iris still visible; and 4 = complete opacity with iris not visible. Mice exhibiting cornea perforation were excluded from subsequent analyses.

### Hematoxylin and Eosin Staining

Corneal morphological changes and inflammatory infiltration were evaluated by hematoxylin and eosin (H&E) staining after AB. This assessment focused on quantifying inflammatory cell infiltration and measuring corneal stromal thickness. Each experimental group included a minimum of eight mouse corneas. Cornea were fixed in 4% paraformaldehyde (PFA), processed for paraffin embedding, and sectioned at 7 µm. Sections were stained with H&E using standard histological protocols. Inflammatory cell counts were conducted on the largest cross-sectional area of the cornea. For each cornea, at least three non-overlapping sections from a single paraffin block were selected to ensure representative and reliable quantification.

### Immunohistochemistry Staining

Paraffin-embedded 7-µm sections were prepared from eyeballs fixed in 4% paraformaldehyde. Sections underwent peroxidase blockade with 3% H_2_O_2_ and microwave-assisted antigen retrieval in buffer before further processing. Sections were blocked with serum and subsequently incubated overnight at 4°C with primary antibodies (YBX1 at 1:50, CD31 at 1:100). After washing, secondary antibodies were applied for 1 hour at room temperature (RT). Signal development was achieved using 3,3ʹ-diaminobenzidine (DAB) as the chromogen. We assessed staining intensity by examining three randomly chosen high-power fields per section using light microscopy. Positive cell counts were obtained via ImageJ software (National Institutes of Health, Bethesda, MD, USA).

### Immunofluorescence Staining

Fixed and dehydrated eyeballs were optimal cutting temperature (OCT) compound-embedded and sectioned in a cryostat. Sections underwent 1-hour blocking with 5% BSA at RT. For cellular immunofluorescence (IF), human umbilical vein endothelial cells (HUVECs) were cultured on glass coverslips placed in petri dishes. After reaching appropriate confluency, cells were rinsed twice with PBS after medium removal. Samples were fixed in 4% PFA (at RT for 30 minutes), permeabilized with 0.5% Triton X-100 (10 minutes), and blocked using 5% BSA for 1 hour at RT. Overnight incubation with specific primary antibodies at 4°C was followed by three PBST washes. Samples were then exposed to fluorescent secondary antibodies for 1 hour at RT, stained with DAPI, and mounted in antifade medium for fluorescence microscopy (Olympus BX53, Japan).

### Western Blotting Analysis

Fresh cornea were homogenized using a tissue grinder, and total protein was extracted from both cornea tissue and HUVECs using a commercial protein extraction kit (R0018; Beyotime, Shanghai, China). Using a BCA protein assay kit (Beyotime, P0010), protein levels were quantified. Aliquots containing 40 µg of protein per lane were electrophoresed on 10% SDS-PAGE gels and then electrotransferred to PVDF membranes in a semi-dry system. Following transfer, membranes were blocked in 5% non-fat milk (1 hour at RT) before overnight incubation at 4°C with the relevant primary antibodies: YBX1 (1:10000), VEGFA (1:500), HIF-1α (1:500), NLRP3 (1:500), NSUN2 (1:1000), ALYREF (1:1,000), CD31 (1:500), Bcl-2 (1:2000), Bax (1:1000), Cleaved PARP (1:1000), Cleaved Caspase-3 (1:500), phospho-JAK1 (p-JAK1, 1:1000), JAK1 (1:1000), phospho-STAT3 (p-STAT3, 1:1000), and STAT3 (1:1000). Following three washes with TBST, membranes were exposed to HRP-linked secondary antibodies for 1 hour at RT. Protein bands were detected using enhanced chemiluminescence substrate. Quantification was carried out using ImageJ software. The uncropped images of Western blot results are showed in the Supplementary Figure S5.

### Cell Culture and Treatment

Acute oxidative stress triggers the inflammation and the development of CoNV following AB.^34^ To mimic the hypoxic microenvironment contributing to CoNV pathogenesis, a hypoxia/reoxygenation (H/R) model was established in vitro. HUVECs (ATCC, Manassas, VA, USA) were maintained in DMEM enriched with 10% fetal bovine serum (FBS), penicillin (100 U/mL), and streptomycin (100 µg/mL), and kept at 37°C in a humidified 5% CO_2_ environment.

Once density reached approximately 80%, HUVECs were switched to DMEM without glucose and serum, and placed in a hypoxic incubator tri-gas incubator (Thermo Scientific Forma II Series, Waltham, MA, USA) under 1% O_2_, 94% N_2_, and 5% CO_2_ at 37°C for 6, 9, or 12 hours. Following hypoxia, complete DMEM was added, and cells were returned to normoxic conditions (5% CO_2_, 95% air) for 24 hours to establish an H/R injury model. Cells maintained under standard normoxic conditions (37°C, 5% CO_2_, 95% air) throughout the experiment served as the control group.

### Cell Viability Assay

Cells were seeded in 96-well plates (5 × 10³ cells/well) and treated for 24 hours with SII (0.1, 1, 5, and 10 µM) or DMSO vehicle, followed by H/R treatment. A CCK-8 assay (Biosharp, BS350A) was used to evaluate viability, with absorbance measured at 450 nm on a Thermo Fisher Scientific microplate reader. Triplicate measurements were normalized to the control condition.

### Small Interfering RNA and Plasmid Transfection

At a density of 1.25 × 10^6^ cells/well in 6-well plates, HUVECs were cultured to 70% to 80% confluence. Transfection with siRNA (100 µM) or overexpression plasmid (5 µg) used Lipofectamine 3000 (supplemented with P3000 for plasmids) in antibiotic-free, serum-free DMEM. Following 24 hours of incubation, medium was changed to complete DMEM. Transfection efficiency was evaluated by Western blot at 72 hours post-transfection.

### Lentiviral Infection

HIF-1α overexpression was achieved by lentiviral transduction. HUVECs plated at 1.25 × 10^6^ cells/well in 6-well plates were infected at 40% to 50% confluence (multiplicity of infection [MOI] = 30, plus 4 µg/mL polybrene). After 24 hours, fresh medium was applied. Puromycin (2.5 µg/mL) was added at 48 hours for selection. Cells were collected 72 hours post-transduction, and HIF-1α expression was verified by Western blot.

### Scratch Wound Healing Assay

After seeding 1 × 10^5^ HUVECs per well in 6-well plates, a scratch wound was created with a sterile 200 µL tip upon reaching confluence. Following gentle PBS washes, low-serum medium (1% FBS in DMEM) was added to minimize proliferation. Migration was monitored over 24 hours under standard culture conditions, with images captured at 0 and 24 hours via phase-contrast microscopy.

### Transwell Migration Assay

Migration was evaluated using transwell inserts (8-µm pores). Cells (4 × 10^5^ per well) in low-serum medium (1% FBS/DMEM) were placed in the upper chamber, the lower chamber contained 500 µL of 10% FBS/DMEM. Post-incubation (24 hours, 37°C), the upper membrane surface was swabbed. Migrated cells were fixed with 3% PFA, stained with 1% crystal violet for 10 minutes, rinsed, and visualized by light microscopy.

### Tube Formation Assay

Matrigel-coated (354234; Corning, NY, USA) 96-well plates were seeded with 2 × 10^4^ HUVECs per well and incubated at 37°C for 4 hours. Images were acquired with an inverted phase-contrast microscope (Olympus BX53, Japan). Quantification analysis was performed by ImageJ software.

### Measurement of Intracellular Reactive Oxygen Species Production

A reactive oxygen species (ROS) detection kit (S0033S; Beyotime, Shanghai, China) was used to quantify oxidative stress. After plating HUVECs at 2 × 10^6^ cells/well in 6-well plates, cells were treated with 20 µM DCFH-DA for 30 minutes (37°C in the dark), washed thoroughly, and immediately imaged with an inverted fluorescence microscope (Olympus IX71). Appropriate controls were run in parallel, and the entire procedure was performed under light-protected conditions.

### TUNEL Staining Assay

Cell apoptosis was assessed via TUNEL staining (Vazyme, Cat #A112). After fixation (4% PFA for 15 minutes) and TBS washing, antigen retrieval was performed in citrate buffer (60°C for 1 hour). Permeabilization with 0.1% Triton X-100 (5 minutes) and Proteinase K digestion (20 µg/mL, 5 minutes) were followed by equilibration (30 minutes at RT). The TdT reaction mixture was incubated overnight at 4°C under dark conditions. Following thorough washes, nuclei were labeled with DAPI, and slides were mounted for fluorescence imaging.

### RNA Sequencing Analysis

Following TRIzol-based RNA extraction from HUVECs, sample quality was evaluated (NanoDrop 2000 for concentration and purity; Agilent 2100 Bioanalyzer for integrity). The VAHTS Universal V10 kit was used for library preparation. OE Biotech Co., Ltd. (Shanghai) carried out the sequencing and bioinformatics analysis.

### RNA Immunoprecipitation and RIP Sequencing Assay

RIP was carried out with kit from BersinBio (Guangzhou, China). Briefly, equal numbers of HUVECs were lysed in polysome buffer containing protease/RNase inhibitors, and DNA was removed. Samples were split into IP, IgG control, and input groups. The lysates were subjected to immunoprecipitation with anti-YBX1 or control IgG overnight at 4°C. Protein A/G magnetic beads were then added (1 hour at 4°C), washed, and bound RNA was extracted with TRIzol for downstream RT-qPCR and RIP-seq (Wuhan Seqhealth Co., Ltd.).

### m^5^C Methylated RNA Immunoprecipitation Assay

Total RNA from HUVECs (2 × 10^7^ cells/group) was extracted and sonicated to generate fragments. Using the m^5^C MeRIP kit (BersinBio, #5204-2), samples were allocated to input, IP (anti-m^5^C antibody), and IgG control groups. Antibody incubation (4°C for 4 hours) was followed by capture with pre-blocked Protein A/G beads. After washing, RNA was eluted, digested with Proteinase K, and purified by phenol-chloroform extraction. Quantified RNA was reverse-transcribed for qPCR validation of m^5^C enrichment.

### m^5^C MeRIP Sequencing Assay

Seqhealth Technology Co., Ltd. (Wuhan, China) carried out m^5^C MeRIP-seq, library preparation, sequencing, and data processing. After TRIzol extraction and DNaseI digestion, poly(A)+ RNA was enriched (VAHTS beads) and fragmented (ZnCl_2_, 95°C) to approximately 100 nt. Ten percent was kept as input; the remainder was incubated with anti-m^5^C antibody and RNasin (2 hours at 4°C), and then captured with Protein A beads (1 hour at 4°C). Washed complexes were eluted (TRIzol). Libraries were generated using the Seqhealth KCTM kit (incorporating UIDs), amplified, size-selected (200–500 bp), and sequenced on a DNBSEQ-T7 (PE150). Raw sequencing data are pending further analysis and have not been submitted to public databases.

### mRNA Stability Assay

After treatment with actinomycin D (10 µg/mL), RNA was extracted from HUVECs at 0, 2, 4, and 6 hours. RT-qPCR was performed to quantify remaining transcripts over time. Data were normalized to the baseline (0 hours) to derive mRNA decay rates.

### Reverse Transcription–Quantitative Polymerase Chain Reaction 

The cDNA was generated from 1 µg of total RNA (extracted by TRIzol-chloroform) with the ABScript III RT Master Mix (ABclonal, RK20429). The qPCR was carried out with SYBR Green chemistry (ABclonal, RK21203), with β-actin used for normalization. All primers were obtained from Sangon Biotech (see the Table). Cycling conditions were: 95°C for 3 minutes, and then 40 cycles of 95°C (5 seconds), 60°C (30 seconds), and 72°C (1 minute). Relative quantification was based on the 2^–ΔΔCt^ method.

**Table.** Sequence of Primers in qRT-PCR

Primer	Sequence
*VEGFA*	AGGGCAGAATCATCACGAAGT(F)
	AGGGTCTCGATTGGATGGCA(R)
*STAT3*	GCAGCTGACTACACTGGCAGAGA(F)
	ATTGTCCAGCCAGACCCAGAA(R)
*Nlrp3*	GATCTTCGCTGCGATCAACAG(F)
	CGTGCATTATCTGAACCCCAC(R)
*Il1b*	ATGATGGCTTATTACAGTGGCAA(F)
	GTCGGAGATTCGTAGCTGGA(R)
*Tnfa*	GAGGCCAAGCCCTGGTATG(F)
	CGGGCCGATTGATCTCAGC(R)
*Il6*	ACTCACCTCTTCAGAACGAATTG(F)
	CCATCTTTGGAAGGTTCAGGTTG(R)
*β-Actin*	GAAATCGTGCGTGACATCAAA(F)
	TGTAGTTTCATGGATGCCACAG(R)

### Statistical Analysis

All data are presented as the mean ± standard error of the mean (SEM). Inter-group differences were evaluated by 1-way ANOVA (Tukey's post hoc test for multiple comparisons) or by 2-tailed unpaired *t*-test for two-group comparisons. GraphPad Prism 9 was used for statistical calculations, with *P* < 0.05 considered significant.

## Results

### CoNV Was Induced by Alkali Burn in C57BL/6 Mice

**Figure 1.**
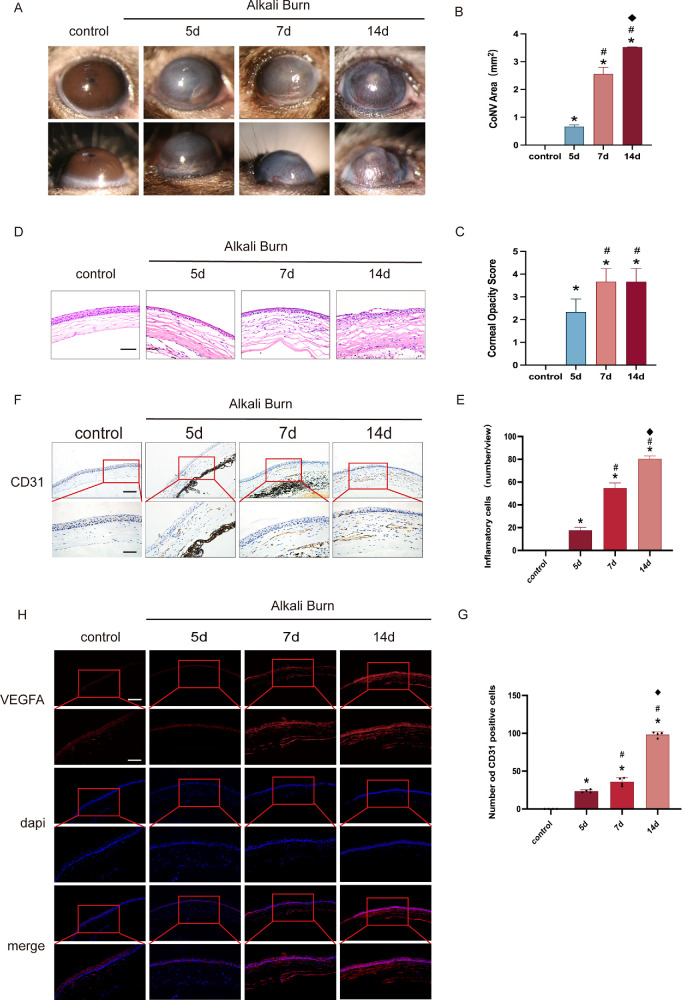
Corneal neovascularization, inflammation, and opacity induced by alkali burn. (**A–C**) Slit-lamp examination showing progressive corneal neovascularization and opacity at days 5, 7, and 14 after alkali burn. (**D, E**) H&E staining reveals increasing stromal edema and inflammatory cell infiltration over time following injury. Magnification = 400×; *scale bar* = 250 µm. (**F, G**) IHC staining for CD31 demonstrates a time-dependent increase in CD31-positive endothelial cells in cornea stroma after alkali burn. Magnification = 200× and 400×; *scale bars* = 500 and 250 µm, respectively. (**H**) IF staining shows gradual upregulation of VEGFA expression, with peak levels observed at day 14. Magnification = 200× and 400×; *scale bars* = 500 and 250 µm, respectively. Data are presented as mean ± SEM (*n* = 8 per group). Means were compared by 1-way ANOVA with Tukey's multiple comparison post hoc tests. *P < 0.05 versus the control group, #*P* < 0.05 versus the 5-day group, ^◆^*P* < 0.05 versus the 7-day group.

The corneas of control mice remained transparent and avascular. Following AB, the corneas exhibited significant inflammation, characterized by stromal edema, opacity, and progressive neovascularization toward the central cornea. Both the vascular area and length increased over time, peaking on day 14 (Figs. 1A–C). H&E staining revealed that stromal edema, detachment of Descemet’s membrane, and inflammatory cell infiltration became more pronounced over the course of the experiment (Figs. 1D, 1E). The endothelial cell marker CD31 was not detected in control corneas. In contrast, the number of CD31^+^ cells increased progressively after injury, forming dense networks in the central cornea by day 14 (Figs. 1F, 1G). Consistent with these findings, immunofluorescence staining demonstrated a time-dependent upregulation of VEGFA expression (Fig. 1H). In summary, the AB effectively induced corneal neovascularization, with the most prominent effects observed on day 14. Therefore, this time point was selected for subsequent mechanistic investigations and therapeutic interventions.

### YBX1 Increased in Cornea after Alkali Burn

**Figure 2.**
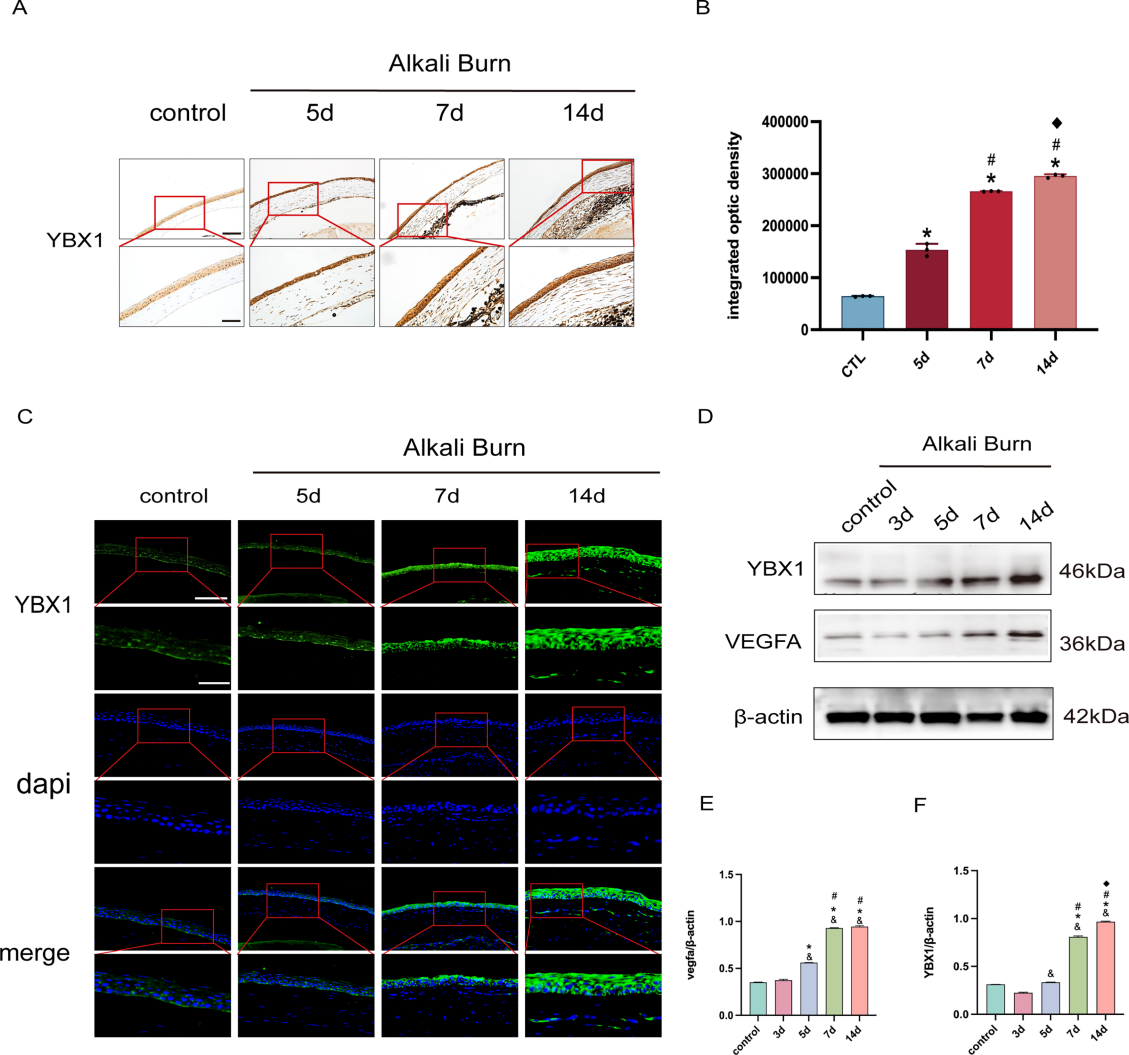
YBX1 increased in the cornea following alkali burn. (**A, B**) IHC staining of YBX1 in the cornea at days 5, 7, and 14 post-alkali burn. Quantification was performed based on integrated optical density (IntDen). Magnification = 200×; *scale bar* = 500 µm, 400×; *scale bar* = 250 µm. (**C**) IF images shows increased YBX1 expression in cornea tissue at days 5, 7, and 14 post-burn. Magnification = 200×; *scale bar* = 250 µm, 400×; *scale bar* = 125 µm. (**D–F**) Western blot results of YBX1 and VEGFA protein expression in cornea at days 3, 5, 7, and 14 after alkali burn, showing time-dependent increases compared to the control group. Data are presented as mean ± SEM (*n* = 8 per group). Means were compared by 1-way ANOVA with Tukey's multiple comparison post hoc tests. *P < 0.05 versus the control group, & *P* < 0.05 versus the 3-day group, #*P* < 0.05 versus the 5-day group, ^◆^*P* < 0.05 versus the 7-day group.

Under normal physiological conditions, YBX1 is primarily localized in the epithelium of the cornea and is expressed at low levels. IHC demonstrated a progressive increase in YBX1 positive staining following AB injury. Quantitative analysis showed that the integrated optical density (IntDen) of YBX1 increased over time, with YBX1 positive cells extending into the stromal layer of the central cornea from the limbus (Figs. 2A, 2B). IF staining revealed that YBX1 fluorescence signals in the cornea epithelium gradually intensified from day 3 to day 14 post-burn. A small number of YBX1 positive cells were also detected in the cornea stroma at later stages (Fig. 2C). Western blot analysis corroborated these observations by showing that the protein levels of YBX1 and VEGFA in the cornea increased progressively at days 3, 5, and 7 following AB, peaking at day 14 (Figs. 2D–F).

### YBX1 May Take Part in Angiogenesis of HUVECs After H/R

**Figure 3.**
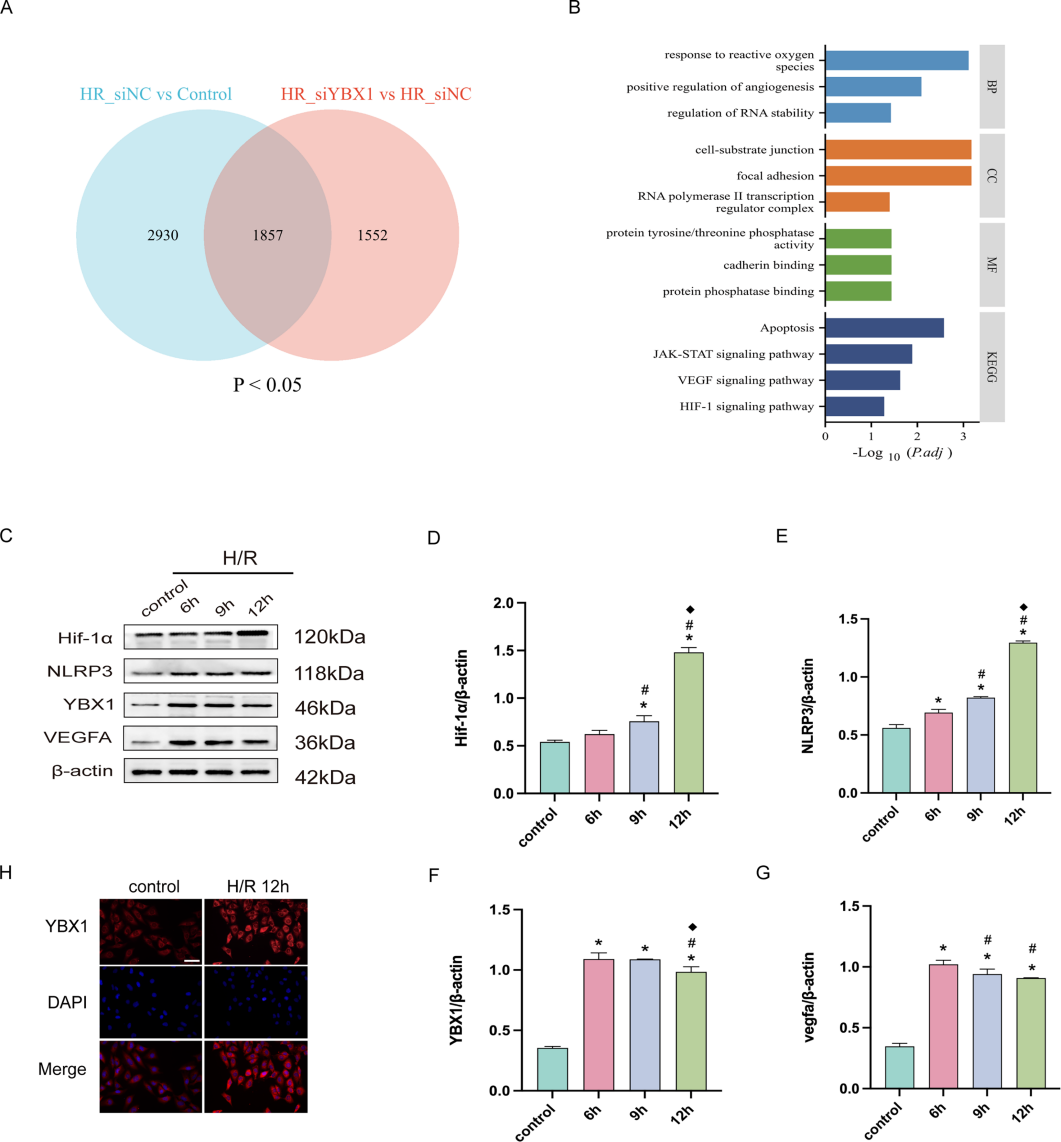
YBX1 could play a role in angiogenesis induced by H/R. (**A,** **B**) Venn diagram showing the intersection of DEGs in the HR_siNC group and the HR_siYBX1 group (*P* < 0.05), and GO and KEGG enrichment analysis of overlapping genes. (**C–G**) WB results and quantitative analysis of YBX1, HIF-1α, NLRP3, and VEGFA expression at different time points after H/R. (**H**) Immunocytochemistry (ICC) staining showing increased YBX1 expression in the cytoplasm of HUVECs after hypoxia. Magnification = 400×; *Scale bar* = 250 µm. Data are presented as mean ± SEM (*n* = 6). Means were compared by 1-way ANOVA with Tukey's multiple comparison post hoc tests. *P < 0.05 versus the control group; #*P* < 0.05 versus the H/R 6-hour group; ^◆^*P* < 0.05 versus the H/R 9-hour group.

Transcriptome sequencing was performed on four groups of HUVECs: the control, HR, and HR_siNC, and HR_siYBX1 (*n* = 3). The intersection of differentially expressed genes (DEGs; *P* < 0.05) from the two comparisons (HR_siNC versus control and HR_siYBX1 versus HR_siNC) yielded 1857 common DEGs (Fig. 3A). Gene ontology (GO) and Kyoto Encyclopedia of Genes and Genomes (KEGG) analyses revealed that these common DEGs were enriched in processes such as response to ROS, positive regulation of angiogenesis, and regulation of RNA stability, and were potentially involved in signaling pathways including apoptosis, JAK-STAT, VEGF, and HIF-1 (Fig. 3B). See Supplementary Figure S2 for detailed differential expression analysis between groups.

Immunoblotting analysis corroborated a marked induction of YBX1 protein following a 12-hour period of hypoxic exposure, relative to normoxic controls, similar to the protein levels of HIF-1α, NLRP3, and VEGFA (Figs. 3C–G). IHC revealed predominant cytoplasmic localization of YBX1 in HUVECs, with elevated expression after 12 hours of hypoxia (Fig. 3H).

### Inhibition of YBX1 Decreased Angiogenesis in HUVECs

**Figure 4.**
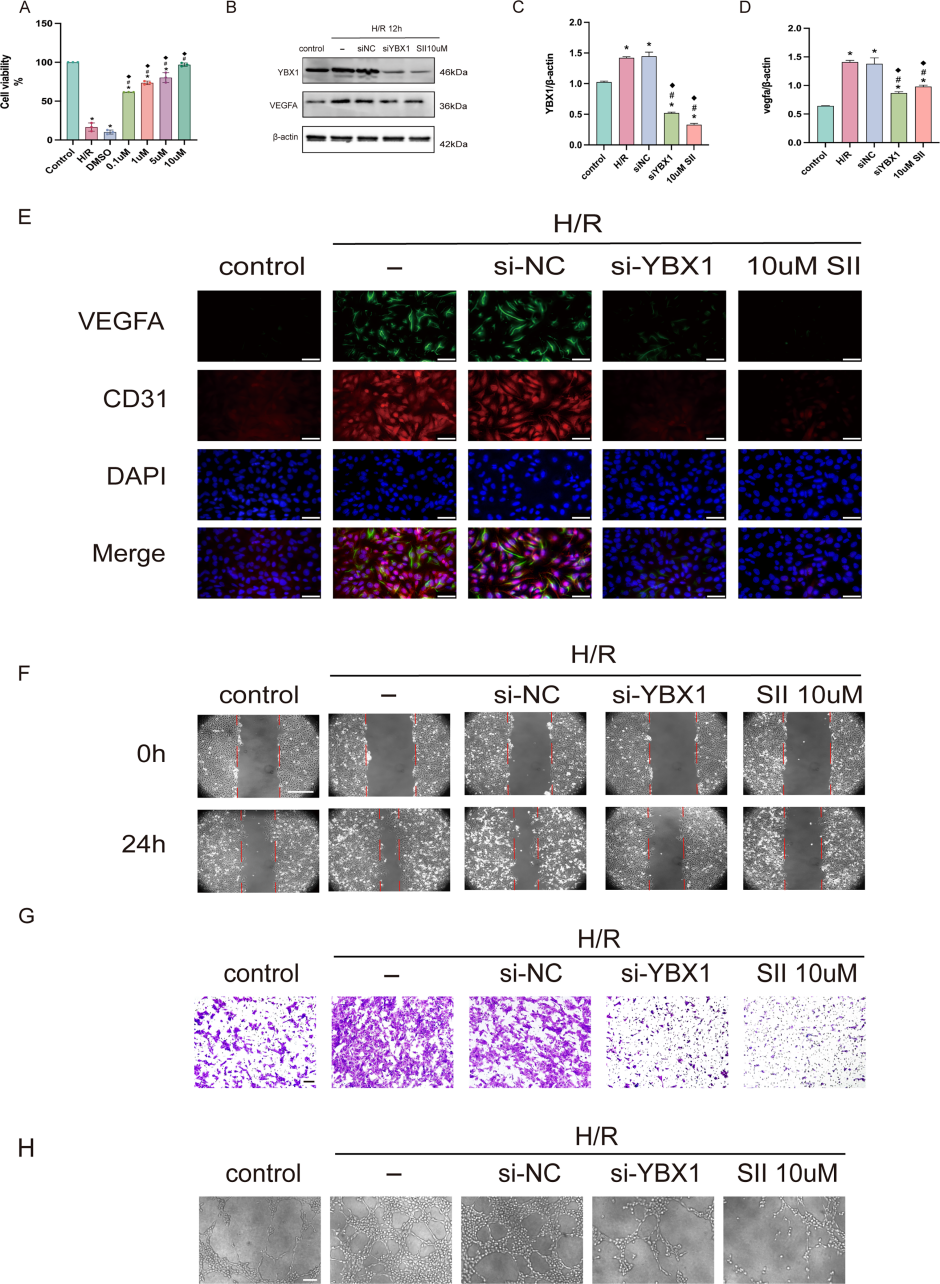
Inhibition of YBX1 in HUVECs resulted in decreased angiogenesis after H/R. (**A**) CCK-8 assay assessing cell viability at different concentrations of soyasaponin II. *P < 0.05 compared to the control group, #*P* < 0.05 compared to the H/R group, ^◆^*P* < 0.05 compared to the H/R group + DMSO group. (**B–D**) Western blot shows that VEGFA expression was significantly inhibited by siYBX1 and 10 µM SII. *P < 0.05 versus the control group, #*P* < 0.05 versus the H/R group, ^◆^*P* < 0.05 versus the H/R + siNC group. (**E**) ICC double staining of VEGFA and CD31 in HUVECs after H/R and treatment. Magnification = 400×; *Scale bar* = 50 µm. (**F**) Scratch wound healing assay showing the ability of HUVECs migration among groups. Magnification = 200×; *Scale bar* = 250 µm. (**G**) Transwell migration assay evaluating the ability of HUVEC invasion among groups. Magnification = 200×; *Scale bar* = 250 µm. (**H**) Tube formation assay assessing the angiogenic capacity of HUVECs among groups. Magnification = 100×; *Scale bar* = 100 µm. Data are presented as mean ± SEM (*n* = 6). Means were compared by 1-way ANOVA with Tukey's multiple comparison post hoc tests.

SII is a phospho-inhibitor of YBX1 and can simultaneously suppress YBX1 protein expression.^35^ A CCK-8 assay was conducted to determine the optimal drug concentration, and results showed that cell viability peaked at 10 µM (Fig. 4A). Therefore, this concentration was selected for subsequent experiments. After inhibiting YBX1 in HUVECs with siRNA or SII (10 µM for 24 hours) followed by 12 hours of hypoxia, we observed that VEGFA expression was significantly reduced (Figs. 4B–D). H/R-induced elevation of CD31 and VEGFA was also reversed by YBX1 inhibition (Fig. 4E). Cell migration, invasion, and tube formation were all notably attenuated upon YBX1 knockdown or SII treatment (Fig. 4F–H). Quantitative analysis results are presented in Supplementary Figures S3A to S3D).

### YBX1 Regulated HIF-1α/NLRP3/Apoptosis/JAK1/STAT3 and ROS in HUVECs After H/R

**Figure 5.**
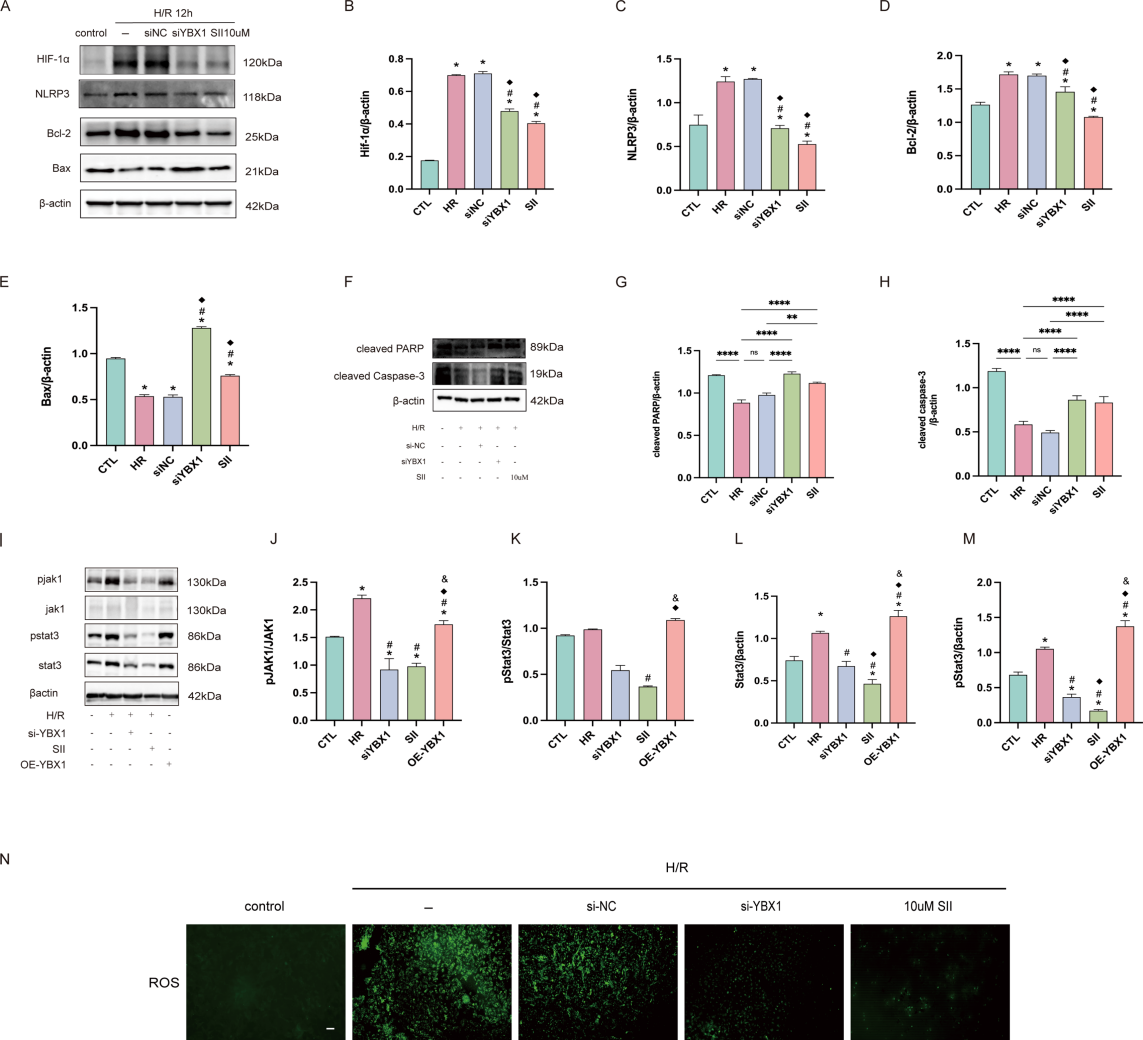
YBX1 regulates HIF-1α/NLRP3/apoptosis/JAK1/STAT3 and ROS in HUVECs after H/R. (**A–H**) Western blot results and quantitative evaluation of HIF-1α, NLRP3, and apoptosis-related proteins (Bcl-2, Bax, cleaved PARP, and cleaved Caspase-3) in each treatment group. *P < 0.05 versus the control group, #*P* < 0.05 versus the H/R group, ^◆^*P* < 0.05 versus the H/R + siNC group. (**I–M**) Western blot results and quantification of JAK1, STAT3, and their phosphorylated forms, as well as pSTAT3/STAT3 and pJAK1/JAK1 ratios across groups. *P < 0.05 versus the control group, #*P* < 0.05 versus the H/R group, ^◆^*P* < 0.05 versus the H/R + siYBX1 group, & *P* < 0.05 versus the H/R + SII group. (**N**) DCFH-DA staining at a probe concentration of 20 µM indicates the level of intracellular ROS production. Magnification = 100×; *Scale bar* = 500 µm. Data are presented as mean ± SEM (*n* = 6). Means were compared by 1-way ANOVA with Tukey's multiple comparison post hoc tests.

After 12 hours of hypoxia, protein levels of HIF-1α, NLRP3, and Bcl-2 rose in HUVECs, whereas Bax, cleaved PARP, and cleaved Caspase-3 fell. Inhibition of YBX1 (by siRNA) or addition of 10 µM SII significantly reversed these trends (Figs. 5A–H).

Moreover, levels of pJAK1, JAK1, pSTAT3, and STAT3 rose with hypoxia or YBX1 overexpression, and fell after YBX1 silencing or SII treatment. The pSTAT3/STAT3 ratio, however, showed no significant change (Figs. 5I–M).

Aditionally, 2′,7′-DCFH-DA staining showed that ROS levels were markedly elevated after hypoxia exposure, and significantly reduced following YBX1 knockdown or SII treatment (Fig. 5N).

### YBX1 Regulates Angiogenesis and Apoptosis Through the HIF-1α Pathway

**Figure 6.**
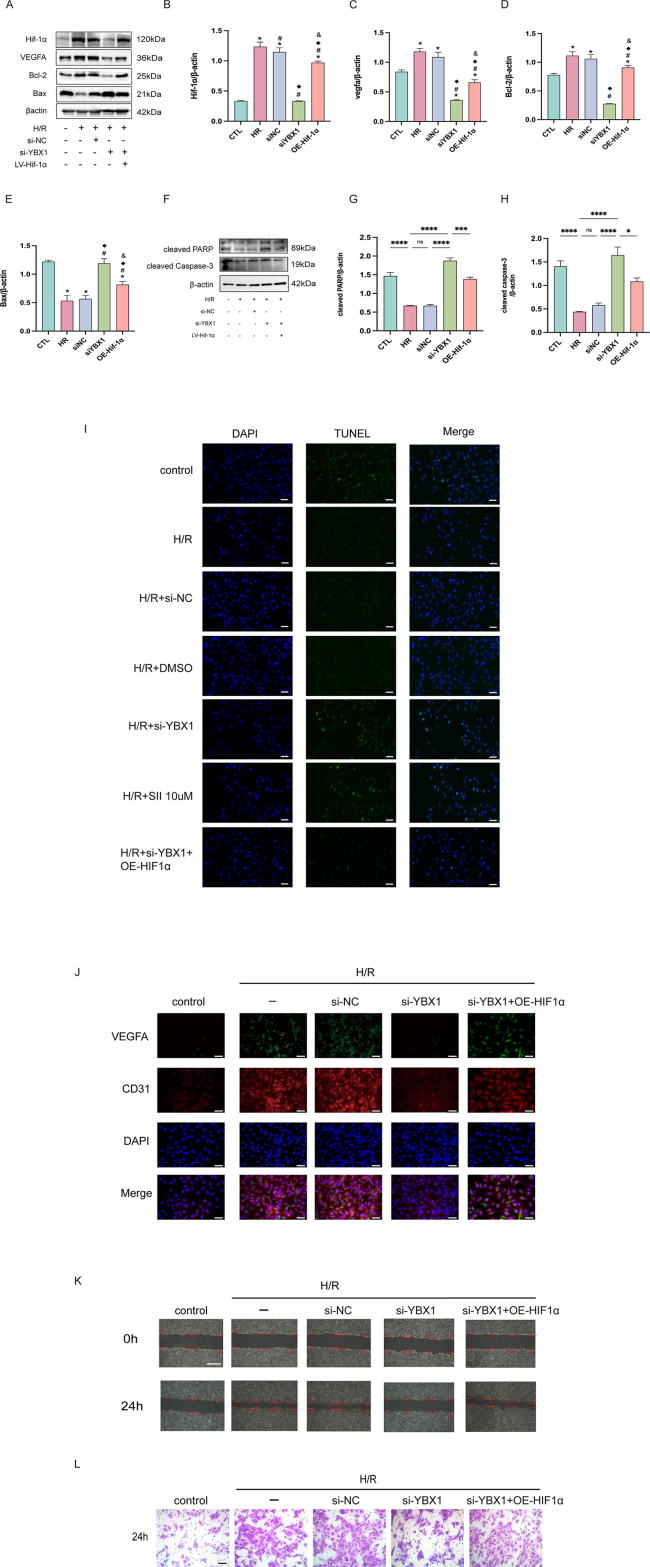
YBX1 regulates angiogenesis and apoptosis in HUVECs through the HIF-1α pathway. (**A–H**) Western blot results and quantification of HIF-1α, VEGFA, Bcl-2, Bax, cleaved PARP, and cleaved Caspase-3 expression levels after overexpression of HIF-1α in the context of YBX1 knockdown. (**I**) TUNEL staining of control, H/R and different treatment groups evaluating by fluorescence microscope. Magnification = 40×; *Scale bar* = 50 µm. (**J**) Double ICC labeling for VEGFA and CD31 in HUVECs with YBX1 knockdown and HIF-1α overexpression. Magnification = 400×; *Scale bar* = 50 µm. (**K**) Scratch wound healing assay showing changes in cell migration ability among different treatment groups. Magnification = 100×; *Scale bar* = 500 µm. (**L**) Transwell migration assay assessing invasion capacity after YBX1 knockdown and HIF-1α overexpression. Magnification = 400×; *Scale bar* = 125 µm. *P < 0.05 versus the control group, #*P <* 0.05 versus the H/R group, ^◆^*P <* 0.05 versus the H/R + siNC group, & *P <* 0.05 versus the H/R + siYBX1. Data are presented as mean ± SEM (*n* = 6). Means were compared by 1-way ANOVA with Tukey's multiple comparison post hoc tests.

To determine whether YBX1 regulates angiogenesis through HIF-1α, a rescue experiment was conducted. A stable HUVECs line was generated by transducing cells with lentivirus expressing HIF-1α, while simultaneously knocking down YBX1 using siRNA. YBX1 knockdown lowered HIF-1α, VEGFA, and Bcl-2 protein level, raised Bax, cleaved PARP, and cleaved Caspase-3, as shown by Western blot. Overexpressing HIF-1α counteracted these changes (Fig. 6A–H). The results of TUNEL staining in HUVECs were consistent with the apoptosis levels detected by Western blotting (Fig. 6I).

HIF-1α overexpression restored VEGFA and CD31 levels downregulated by YBX1 knockdown, as evidenced by immunocytochemistry (ICC) double staining (Fig. 6J). Wound healing and transwell migration assays further indicated that HIF-1α overexpression rescued the migration and invasion deficits caused by YBX1 knockdown (Figs. 6K, 6L). See Supplementary Figures S3E and S3F for quantitative analysis.

### YBX1 Affects mRNA Stability of STAT3 and VEGFA as an m^5^C Reader

**Figure 7.**
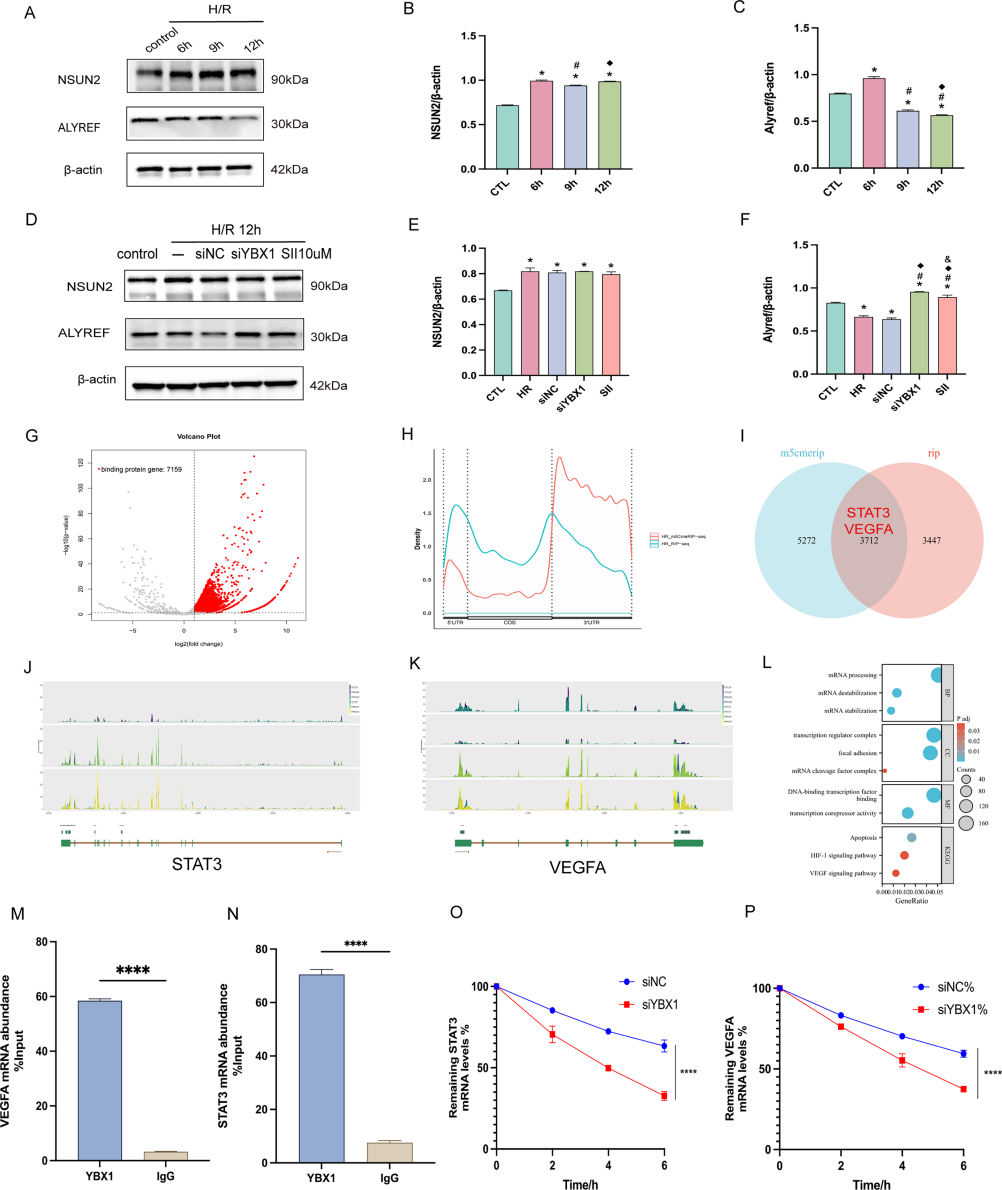
YBX1 impacted mRNA stability of STAT3 and VEGFA as an m^5^C RNA binding protein under hypoxia in HUVECs. (**A–C**) NSUN2 and Alyref protein levels were elevated and decreased, respectively, in the H/R 12-hour group compared with the control group, and were quantified and analyzed. *P < 0.05 versus the control group, #*P <* 0.05 versus the H/R 6-hour group, ^◆^*P <* 0.05 versus the H/R 9-hour group. (**D–F**) Following YBX1 inhibition, NSUN2 levels remained unchanged, whereas ALYREF expression significantly increased. *P < 0.05 versus the control group, #*P <* 0.05 versus the H/R group, ^◆^*P <* 0.05 versus the H/R + siNC group, & *P <* 0.05 versus the HR + siYBX1. (**G**) RIP-seq identified 7159 differentially upregulated genes in the IP group compared to the input group under H/R. (**H**) Distribution of RIP-seq and m^5^C MeRIP-seq peaks across transcriptomic regions. (**I**) Venn diagram showing 3712 intersecting genes between YBX1 binding genes and m^5^C peak calling annotation genes. (**J, K**) Geneplot diagrams show the enrichment of m^5^C modification peaks and YBX1 binding peaks on the STAT3 and VEGFA transcriptions, respectively. (**L**) GO and KEGG pathway enrichment analysis of the intersecting genes. (**M, N**) RIP-qPCR validation of the binding between YBX1 and STAT3 or VEGFA mRNA. (**O, P**) Actinomycin D assay showing that YBX1 knockdown accelerated degradation of STAT3 and VEGFA mRNA. ****P < 0.0001. Data are presented as mean ± SEM (*n* = 6). Means were compared by 1-way ANOVA with Tukey's multiple comparison post hoc tests **B** to **C**, **E** and **F**, and **O** to **P** and 2-tailed unpaired Student's *t*-test **M** and **N**.

Following H/R treatment, NSUN2 expression rose, whereas ALYREF decreased in HUVECs (Figs. 7A–C). YBX1 knockdown or SII treatment left NSUN2 levels unaffected but increased ALYREF expression compared to the H/R + siNC group (Figs. 7D–F).

To identify the downstream targets recognized by YBX1 as an m^5^C reader, RIP-seq and m^5^C MeRIP-seq were performed in HUVECs after H/R, comparing the IP and input groups. See sequencing data quality analysis in Supplementary Figure S4. Volcano plot analysis revealed 7159 significantly upregulated genes in the IP group (Fig. 7G). Meta-gene analysis showed that YBX1-RIP peaks were enriched at the 5′ UTR transcripts, whereas m^5^C signals showed classical enrichment at the 3′ UTR (Fig. 7H). Sequence motif analysis revealed shared m^5^C-related motifs in the two datasets (Supplementary Fig. S4). The overlap between protein-binding genes in ripseq and m^5^C meripseq peak calling annotation genes was intersected to obtain 3712 genes, including STAT3 and VEGFA (Fig. 7I). IGV visualization showed co-localization of YBX1 binding and m^5^C modification signals at STAT3 and VEGFA loci (Figs. 7J, 7K). GO and KEGG analysis demonstrated that these overlapping targets were strongly linked to key pathways, including mRNA processing, mRNA stabilization, focal adhesion, apoptosis, HIF-1α signaling, and VEGF signaling (Fig. 7L). RIP assays further indicated the direct interaction between YBX1 and the mRNAs of STAT3 and VEGFA (Figs. 7M, 7N). Actinomycin D treatment showed that depleting YBX1 accelerated the degradation of STAT3 and VEGFA mRNA (Figs. 7O, 7P).

### m^5^C Modification Is Required for the YBX1-STAT3/VEGFA mRNA Interaction

**Figure 8.**
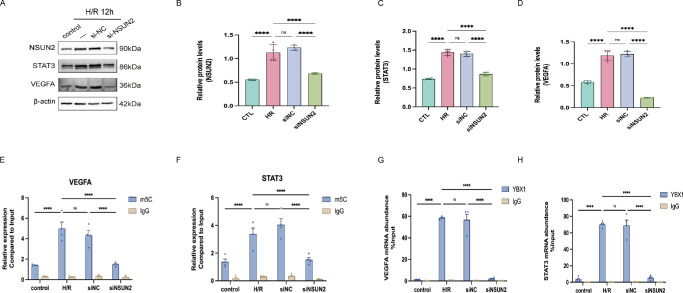
NSUN2-mediated m^5^C modification is required for the YBX1-STAT3/VEGFA mRNA interaction. (**A–D**) Knockdown of NSUN2 by siRNA resulted in a significant reduction in the protein levels of both STAT3 and VEGFA. (**E, F**) The m^5^C Merip-qPCR analysis demonstrating the m^5^C enrichment levels on VEGFA and STAT3 mRNA across different treatment groups. (**G,** **H**) Rip-qPCR analysis of the binding of YBX1 to VEGFA and STAT3 mRNAs in different treatment groups. Means were compared by 1-way ANOVA with Tukey's multiple comparison post hoc tests. *P < 0.05, **P < 0.01, ***P < 0.001, ****P < 0.0001, ns represents no significant difference.

Knockdown of the methyltransferase NSUN2 resulted in reduced STAT3 and VEGFA protein levels (Figs. 8A–D). Concomitantly, m^5^C-MeRIP confirmed a significant decrease in m^5^C enrichment on STAT3 and VEGFA transcripts upon NSUN2 depletion (Figs. 8E, 8F). Furthermore, RIP-qPCR demonstrated that the binding of YBX1 to these mRNAs was diminished following NSUN2 knockdown (Figs. 8G, 8H).

### SII Alleviated Inflammation and Neovascularization in Mice Cornea After Alkali Burn

**Figure 9.**
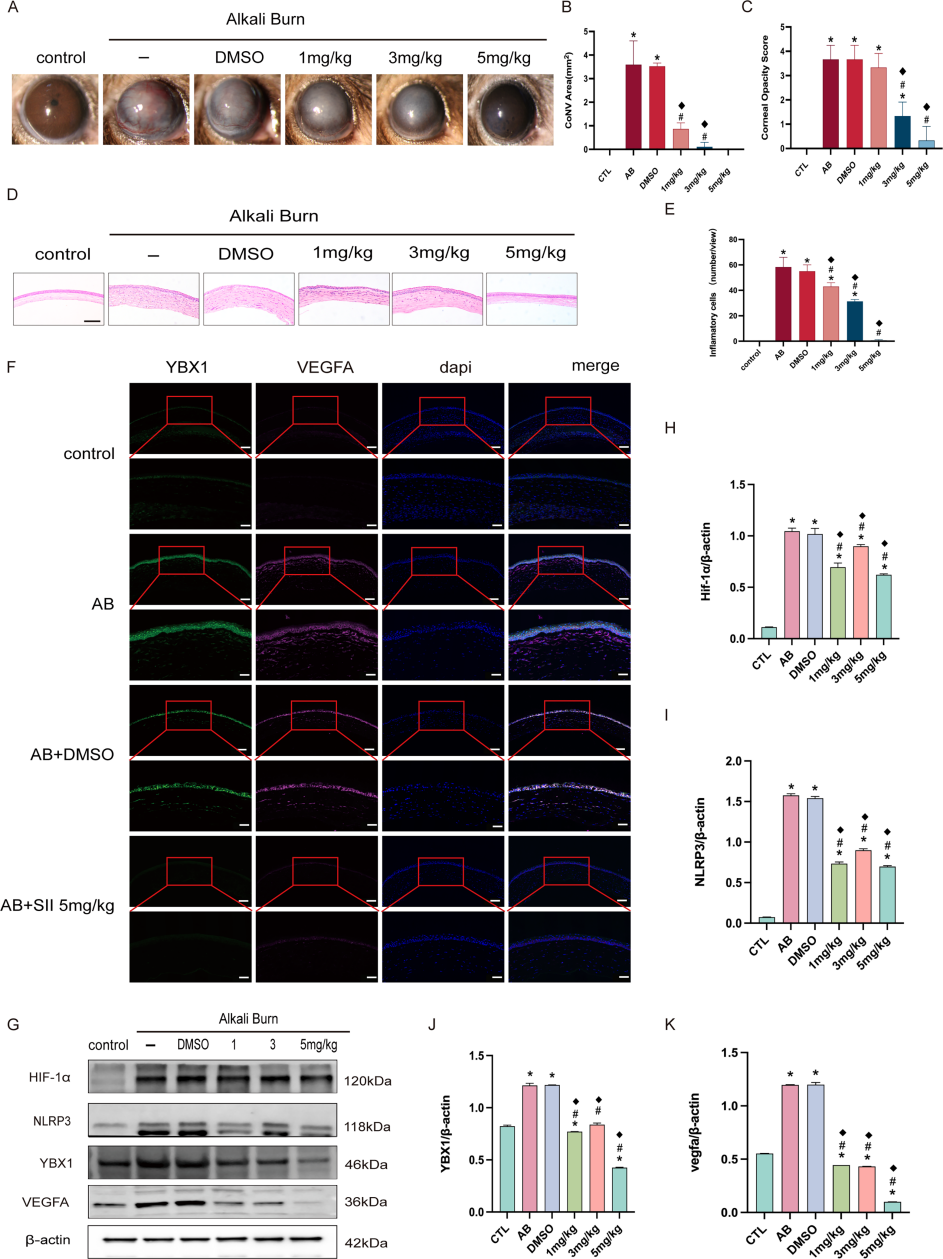
Treatment with SII alleviated corneal neovascularization and inflammation induced by alkali burn. (**A–C**) Slit-lamp images of mouse corneas and quantitative analysis of neovascularization and transparency in groups treated with different drug concentrations. (**D, E**) H&E staining and quantification of inflammatory cell in cornea among groups. Magnification = 200×; *Scale bar* = 500 µm. (**F**) Double immunofluorescence labeling for YBX1 and VEGFA in cornea from each group. Magnification = 200×; *Scale bar* = 50 µm; Magnification = 400×; *Scale bar* = 25 µm. (**G–K**) Western blot results of related proteins in cornea following in vivo drug administration, along with quantitative analysis. Data are presented as mean ± SEM (*n* = 8). Means were compared by 1-way ANOVA with Tukey's multiple comparison post hoc tests. *P < 0.05 versus the control group, #*P <* 0.05 versus the AB group, ^◆^*P <* 0.05 versus the AB + DMSO group.

Relative to AB and vehicle-treated corneas, SII dose-dependently reduced neovascularization and improved corneal transparency, achieving optimal outcomes at 5 mg/kg (Figs. 9A–C). Decreasing corneal stromal edema and the number of inflammatory cells caused by SII were shown in H&E results (Figs. 9D, 9E). Double IF staining revealed the markedly reduced level of YBX1 and VEGFA following SII administration (Fig. 9F). Significantly downregulated protein levels of HIF-1α, NLRP3, and VEGFA induced by SII were also confirmed by Western blot (Figs. 9G–K).

### SII Modulated Macrophage and Reduced Proinflammatory Factor Release

**Figure 10.**
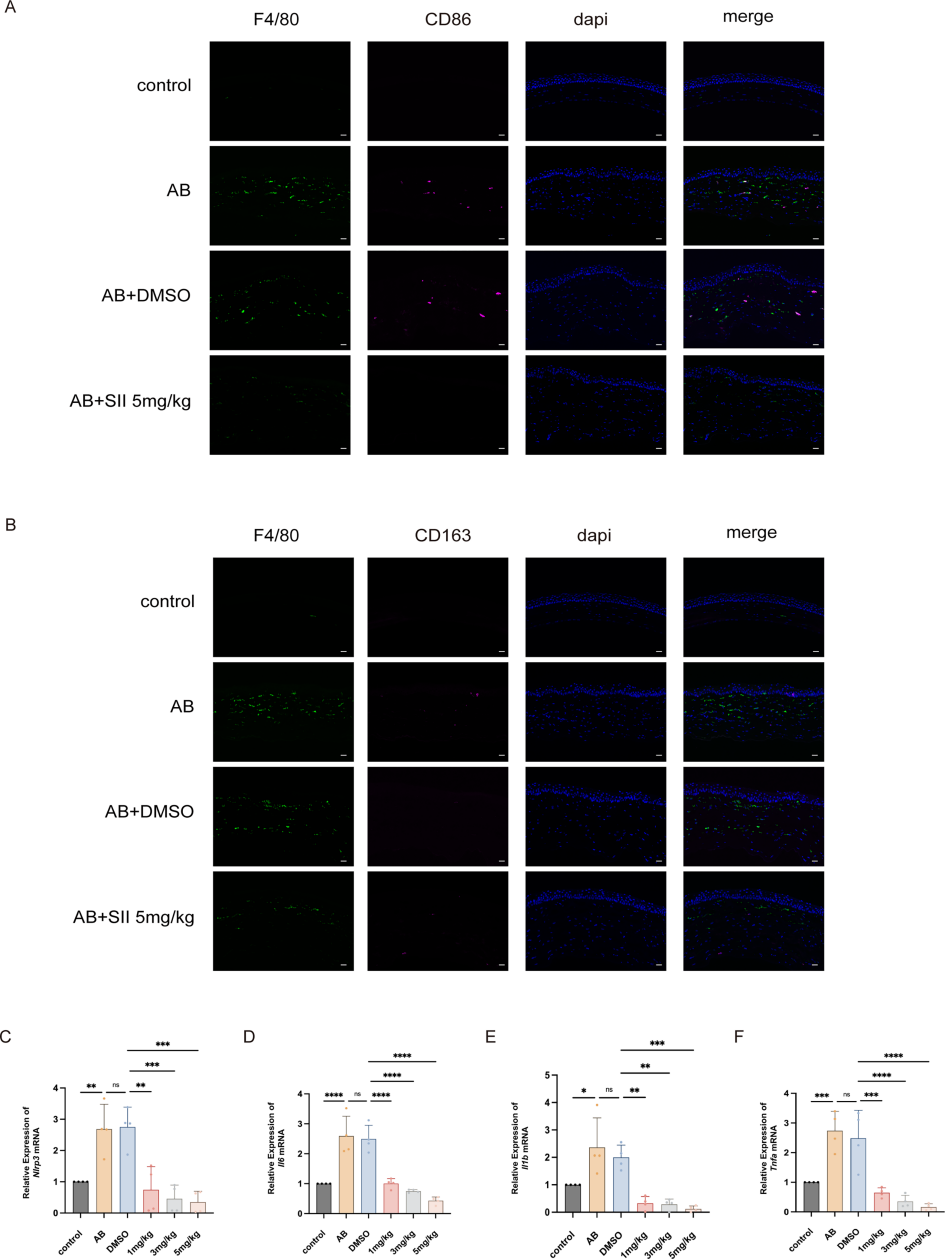
SII modulated macrophage polarization and suppressed proinflammatory factors. (**A**) CD86 and F4/80 double-stained unpolarized and M1 polarized macrophages in the cornea. (**B**) CD163 and F4/80 double-stained unpolarized and M2 polarized macrophages in the cornea. Magnification = 400×; *Scale bar* = 50 µm. (**C**–**F**) The qPCR analysis of the mRNA levels of pro-inflammatory cytokines in the mouse cornea. Means were compared by 1-way ANOVA with Tukey's multiple comparison post hoc tests. *P < 0.05, **P < 0.01, ***P < 0.001, ****P < 0.0001, ns represents no significant difference.

Macrophage infiltration and polarization were assessed by IF double staining. On day 14 post-AB, we observed an increased number of CD86^+^F4/80^+^ cells in the corneal stroma, with clear co-localization of CD86 and F4/80 (Fig. 10A). Notably, there was no apparent CD163^+^ staining in the stroma (Fig. 10B). The population of CD86+/F4/80+ cells and the total F4/80+ cells were significantly lowered following treatment with 5 mg/kg SII (see Fig. 10A). Concurrently, a small number of CD163^+^ cells became detectable in the stroma (see Fig. 10B).

The mRNA expression of *Nlrp3*, *Il6*, *Il1b*, and *Tnfa* was increased after AB, as shown by qPCR. No significant difference was observed with DMSO, but SII treatment produced a concentration‑dependent decrease in their levels (Figs. 10C–F).

## Discussion

AB, a leading cause of blindness, triggers a cascade of pathological changes, including corneal edema, opacity, inflammatory response, and neovascularization.^3^ Inhibiting angiogenesis and reducing inflammation are crucial strategies to preserve corneal transparency and prevent vision loss. The current treatment options for corneal neovascularization, primarily local corticosteroid-based anti-inflammatory therapy, and corneal transplantation, are hampered by suboptimal efficacy and significant adverse effects.^5^^,^^7^ YBX1 functions as a versatile nucleic acid-binding protein, interacting with both DNA and RNA to modulate gene expression across transcriptional and post-transcriptional levels.^10^ Research has demonstrated its pivotal involvement in modulating proliferative responses and angiogenic processes.^13^^,^^17^^,^^18^ As a classical m^5^C RNA modification reader, YBX1 mediates RNA metabolic events, such as splicing and mRNA stabilization, thereby exerting post-transcriptional control over gene expression.^19^^,^^22^ We initially examined how YBX1 expression is altered in the cornea of alkali-burned mice across a defined time course and, for the first time, performed m^5^C MeRIP-seq in HUVECs to investigate its functional targets. The objective was to determine whether YBX1 modulates CoNV after AB by regulating downstream genes at the post-transcriptional level through recognition of m^5^C-modified mRNAs.

The observed co-upregulation of YBX1, VEGFA, and CD31 in alkali-burned corneas of C57 mice implies a potential role for YBX1 in VEGFA-related pathways during neovascularization, consistent with prior reports.^36^ Evidence from earlier work suggests a role for YBX1 in upregulating key angiogenic factors IL-8, MMP-9, VEGFA, and Ang-1 in HUVECs,^37^ however, are still poorly understood. Our transcriptomic profiling of HUVECs subjected to H/R indicated that YBX1 is involved in the HIF-1, VEGFA, and JAK/STAT signaling pathways, and may be associated with RNA metabolic, apoptosis processes.

In subsequent in vitro experiments, we were surprised to discover that YBX1 promoted migration and angiogenesis in HUVECs following H/R. Li et al. confirmed that shRNA knockdown of YBX1 inhibited HUVEC migration and tube formation, which may be partially attributed to YBX1 knockdown disrupting CD31 mRNA stability through an m^5^C-dependent mechanism, thereby reducing its protein expression.^15^ This is consistent with our findings that YBX1 knockdown reduces CD31 expression. YBX1 may regulate its downstream effector NLRP3 through promoting mRNA nuclear export and stability^38^ or binding to the promoter region to activate transcription of NLRP3.^39^ Moreover, YBX1 could influence the oxidative stress status in HUVECs by regulating the production of ROS.^40^

We further observed that YBX1, whether introduced exogenously via overexpression or induced endogenously by H/R treatment, activates the JAK1/STAT3 signaling pathway. This activation chiefly stems from upregulated constitutive STAT3 expression rather than a change in its phosphorylation status. Although we have confirmed that YBX1 mediates the activation of JAK1/STAT3 at the protein level in HUVECs under H/R, its subsequent transcriptional regulatory function on downstream genes remains to be validated. This warrants further investigation using techniques such as a STAT3 reporter assay or chromatin immunoprecipitation (ChIP).

In vivo and in vitro analyses revealed that HIF-1α expression paralleled that of YBX1. These observations formed the basis for a hypothesis, positing HIF-1α as a downstream mediator of YBX1 in angiogenesis. Subsequent rescue studies showed that YBX1 modulates the expression of VEGFA, CD31, and apoptosis-related proteins^41^^,^^42^ through HIF-1α, thereby modulating the migratory and invasive behavior of HUVECs.

Existing literature indicates that YBX1 interacts directly with the mRNA of HIF-1α, promoting its translation and increasing protein expression levels.^25^^,^^43^ We further investigated the regulatory mechanism of YBX1 on HIF-1α in this disease model. As previously described, YBX1 functions as a reader of m^5^C modifications. We hypothesized that it may regulate downstream molecules and biological processes through m^5^C-dependent mechanisms in HUVECs subjected to H/R. Interestingly, we observed that m^5^C methylation levels mediated by NSUN2 may be elevated after H/R, with YBX1 acting as the primary m^5^C reader rather than ALYREF. YBX1 inhibition had no significant effect on NSUN2 levels, likely because NSUN2 functions upstream of YBX1.

Our epigenomic analysis offers a novel perspective on the role of YBX1 in pathological angiogenesis. The finding that YBX1 binding peaks and m^5^C modification sites co-localize at specific genomic loci, particularly on STAT3 and VEGFA mRNAs, suggesting that YBX1 acts as an m^5^C reader in vascular endothelial cells under stress. Upon binding to these transcripts, YBX1 may stabilize them, as supported by the accelerated degradation of STAT3 and VEGFA mRNA following YBX1 knockdown.^44^ Interestingly, we did not observe direct binding between YBX1 and HIF-1α mRNA in HUVECs under H/R, but instead identified STAT3 as a direct YBX1 target. Previous studies have demonstrated that STAT3 can regulate downstream genes in response to HIF-1α signaling.^28^^,^^45^^,^^46^ Therefore, it is plausible that STAT3 might serve as an intermediary in YBX1-mediated regulation of HIF-1α. We plan to inhibit STAT3 in future experiments to investigate further whether the regulatory effect of YBX1 on HIF-1α is mediated through STAT3.

A key finding is the dependence of this mechanism on the m^5^C methyltransferase NSUN2. Upon NSUN2 knockdown, both the m^5^C modification enrichment on STAT3/VEGFA mRNA and the subsequent binding of YBX1 were significantly reduced. These indicate that m^5^C modification is required for the recognition and binding of YBX1 to its target transcripts. Subsequent techniques such as bisulfite sequencing (BS-seq) to map m^5^C sites with high precision is essential, followed by site-directed mutagenesis and rescue assays to conclusively define the functional contribution of individual modified to the cellular phenotype. We are currently pursuing this approach.

CoNV is largely driven by inflammatory processes, wherein macrophages are critically involved. Following recruitment to the cornea after AB, these cells can polarize towards a pro-inflammatory M1 or an anti-inflammatory M2 state.^47^ Cytokines released by M1 macrophages, including TNF-α, IL-1β, IL-6, and VEGFA, directly contribute to CoNV by enhancing endothelial cell proliferation and migration.^48^ Our results of in vivo experiments demonstrate that beyond its direct anti-angiogenic effect through reducing protein level of HIF-1α, NLRP3, and VEGFA, SII also mitigates the overall inflammatory response in the cornea by reducing macrophage infiltration, promoting a polarization switch to the M2, and inhibiting the release of major inflammatory cytokines. This improves the corneal microenvironment and controls the upstream drivers of CoNV. Our findings posit YBX1 as a potential target for managing corneal ABs, whereas recognizing that comprehensive pharmacokinetic, long-term safety, and efficacy studies are indispensable for any future clinical translation of SII. Our current work establishes a foundational framework for understanding the anti-angiogenic mechanisms of SII and positions it as a highly promising candidate for future translational research.

**Figure 11.**
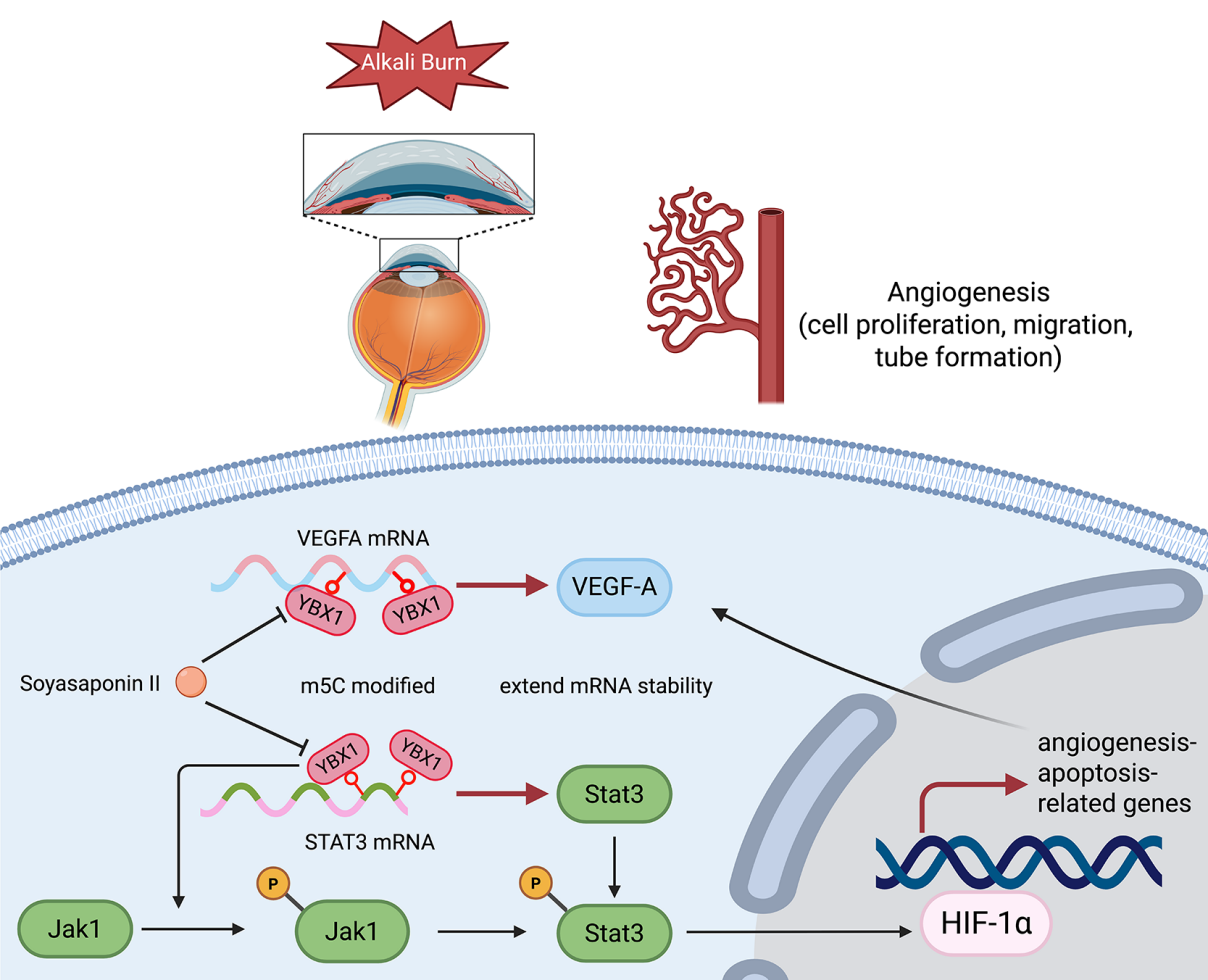
YBX1 might function as a reader of m^5^C modification, influencing angiogenesis and apoptosis in CoNV via the STAT3/HIF-1α/VEGFA pathway.

In conclusion, the study identified YBX1 as an m^5^C reader that post-transcriptionally stabilizes STAT3 and VEGFA mRNA, thereby driving pathological angiogenesis. This stabilization mechanism depends on m^5^C modification, which is catalyzed by NSUN2, and the subsequent essential binding of YBX1 to these modified transcripts. Furthermore, YBX1 likely regulates HUVEC migration, angiogenesis, and apoptosis through the HIF-1α pathway and may also modulate macrophage to promote the inflammatory responses that precede CoNV (Fig. 11). SII, which inhibits YBX1, is expected to be a potential treatment for CoNV.

This study has several limitations. The exact locations of m^5^C modifications could not be determined with nucleotide-level precision due to methodological resolution limits. We agree that single-base mapping is an essential future objective. To address this, precise m^5^C profiling via BS-seq will be performed, after which the contribution of individual sites will be tested functionally through mutation and rescue strategies—an approach now in progress. Second, the corneal microenvironment is a complex multicellular system in which various resident and infiltrating cell types produce VEGFA and other cytokines that influence endothelial cell behavior. To better mimic this physiological setting, future studies should utilize co-culture systems and corneal organoid models to explore how YBX1 regulates interactions among multiple cell types. For the clinical application of the YBX1 inhibitor SII, future studies should prioritize elucidating the pharmacokinetic properties of SII in ocular tissues, conducting detailed dose-ranging studies to optimize its efficacy, and evaluating its long-term benefits and safety profile in more animal models. Moreover, advanced drug delivery methods are vital for successful treatment of the ocular surface. Future efforts will focus on developing biodegradable polymeric nanoparticles for sustained drug release, exosome-based delivery platforms, and CRISPR-Cas9-loaded virus-like particles (VLPs) to achieve precise targeted treatment.^49^^,^^50^

## Conclusions

This research is the first to confirm the regulatory function and mechanism of YBX1 in CoNV. YBX1 modulated angiogenesis and apoptosis by regulating the stability of m^5^C-modified STAT3 and VEGFA mRNAs, thereby activating the JAK1/STAT3/HIF-1α axis. SII may hold therapeutic potential for corneal alkali burns by mitigating CoNV and the inflammation.

## Supplementary Material

Supplement 1
