# Supplementary material for: YBX1 Modulated Corneal Neovascularization Induced by Alkali Burn via m5C-Dependent Regulation of the STAT3/HIF-1α/VEGFA Axis
Source: Invest Ophthalmol Vis Sci. 2026 Feb 23;67(2):42. doi: 10.1167/iovs.67.2.42 (PMC12934531; doi:10.1167/iovs.67.2.42)
Supplement: Supplement 1 [file iovs-67-2-42_s001.pdf]

Table S1. siRNA sequences.

siRNA	Sequence	
siYBX1	Snese	CAAGGAAGAUGUAUUUGUAdTdT
	Antisense	UACAAAUACAUCUUCCUUGdTdT
siNSUN2	Snese	AGCUGUUCGAGCACUACUAdTdT
	Antisense	UAGUAGUGCUCGAACAGCUdTdT
siNC	Snese	UUCUCCGAACGUGUCACGUTT
	Antisense	ACGUGACACGUUCGGAGAATT

Table S2. Antibodies used in this study

Primary Antibodies	Source	Company	Catalog No.	Dilution
YBX1	Rabbit pAb	Proteintech	20339-1-AP	1:1000 WB<br>1:100 IF<br>1:100 ICC<br>1:50 IHC<br>1:100 RIP
VEGFA	Rabbit pAb	Proteintech	19003-1-AP	1:1000 WB<br>1:100 IF<br>1:100 ICC
NSUN2	Rabbit pAb	Proteintech	20854-1-AP	1:20000WB
ALYREF	Rabbit pAb	Proteintech	16690-1-AP	1:1000 WB
β-actin	Rabbit pAb	Proteintech	20536-1-AP	1:10000WB
NLRP3	Rabbit mAb	Abclonal	A24294	1:1000 WB
CD163	Rabbit mAb	abcam	ab182422	1:2000 IF
F4/80	Rabbit mAb	CellSignalingTechnology	70076	1:4000 IF
CD86	Rabbit mAb	CellSignalingTechnology	19589	1:400 IF

Cleaved PARP	Rabbit mAb	CellSignalingTechnology	5625	1:1000 WB
Cleaved Caspase-3	Rabbit mAb	CellSignalingTechnology	9664	1:1000 WB
STAT3	Rabbit mAb	CellSignalingTechnology	4904	1:2000 WB
p-STAT3(Tyr705)	Rabbit mAb	CellSignalingTechnology	9145	1:2000 WB
JAK1	Rabbit pAb	Affinity Biosciences	AF5012	1:1000 WB
p-JAK1 (Tyr1022)	Rabbit pAb	Affinity Biosciences	AF2012	1:1000 WB
HIF1A	Rabbit pAb	Affinity Biosciences	AF1009	1:1000 WB
CD31	Goat pAb	R&D system	AF3628	1:100 ICC<br>1:100 IHC
Bcl-2	Rabbit mAb	HUABIO	ET1702-53	1:5000 WB
Bax	Rabbit mAb	HUABIO	ET1603-34	1:20000WB

**Secondary Antibodies**	**Source**	**Company**	**Catalog No.**	**Dilution**
Goat Anti-Rabbit IgG (H&L) (HRP)	Goat	Proteintech	SA00001-2	1:10000 WB
Donkey Anti-Rabbit IgG (H&L) (Alexa Fluor® 488)	Donkey	Jackson ImmunoResearch	711-545-152	1:1000 IF
Donkey Anti-Rabbit IgG (H&L) (Alexa Fluor® 594)	Donkey	Jackson ImmunoResearch	711-585-152	1:1000 IF
Donkey Anti-Goat IgG (H&L) (Alexa Fluor® 594)	Donkey	Jackson ImmunoResearch	705-585-003	1:100 IF

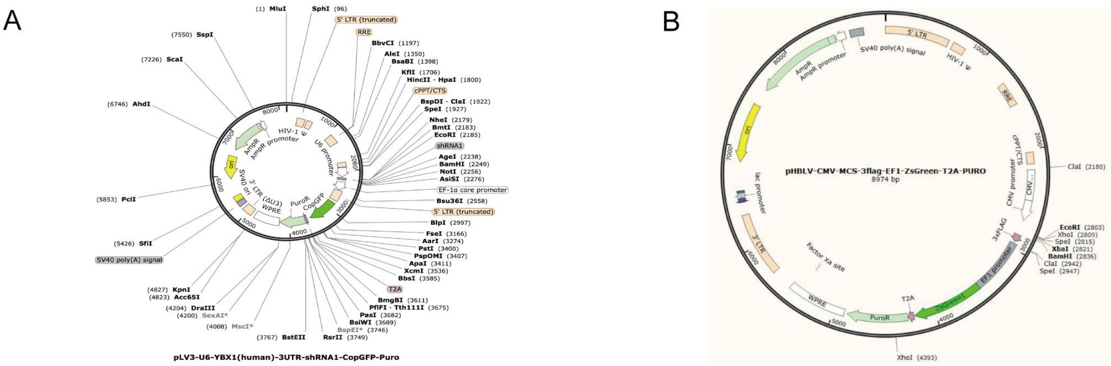

Fig. S1. Plasmid map diagrams used in the study. (A) Plasmid map diagram of pLV3-U6-YBX1(human)-3UTR-shRNA1-CopGFP-Puro. (B) Plasmid map diagram of pHBLV-CMV-MCS-3flag-EF1-ZsGreen-T2A-PURO.

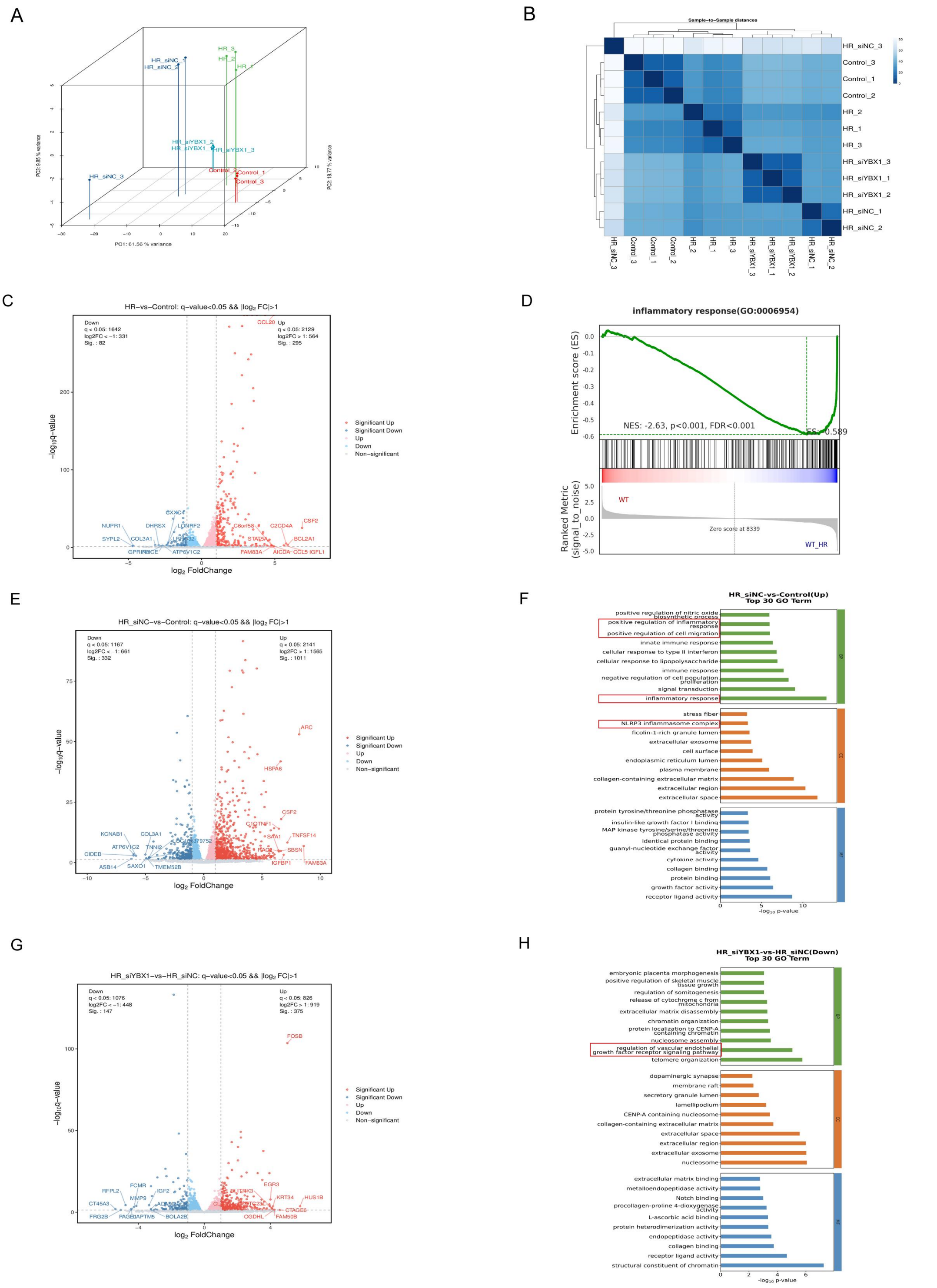

Fig. S2. Principal component and correlation analysis of transcriptome sequencing data samples across groups. (A) Principal component analysis (PCA) showed clear separation among the four groups. (B) Correlation heatmap demonstrated high intra-group consistency. (C, D) Volcano plot analysis revealed 295 significantly upregulated and 82 downregulated genes in the H/R group compared to the control group and Gene Set Enrichment Analysis (GSEA) indicated that these genes involved in the inflammatory response (NES = –2.63, $P < 0.001$) were significantly enriched in the H/R group. (E, F) Compared to the Control group, 1011 genes were upregulated in the HR_siNC group ($q < 0.05$, $|\log_2 FC| > 1$) and enriched in biological processes including positive regulation of inflammatory response, cell migration and NLRP3 inflammasome complex. (G, H) 147 genes were downregulated in the HR_siYBX1 group relative to the HR_siNC group ($q < 0.05$, $|\log_2 FC| > 1$), which were enriched in the regulation of the vascular endothelial growth factor receptor signaling pathway.

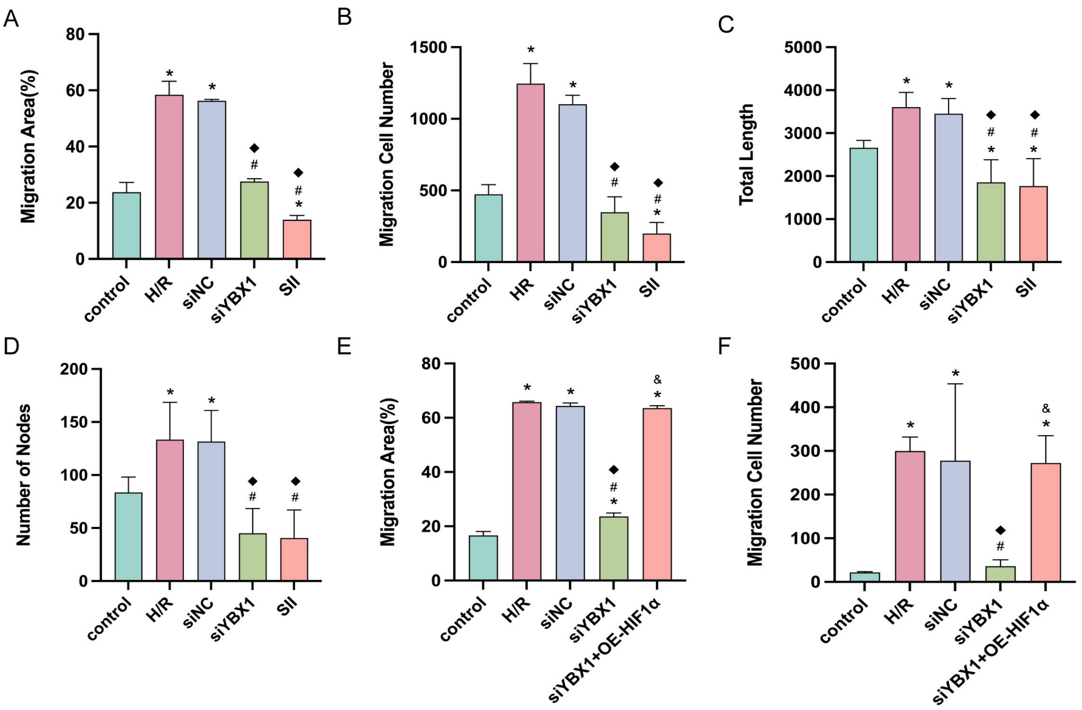

Fig. S3. Quantitative analysis of results of HUVECs phenotypic experiments. (A, E) Quantitative analysis of migration area in scratch wound healing assays. (B, F) Quantitative analysis of migration cell number in transwell assays. (C, D) Quantitative analysis of total length and number of nodes in tube formation assay.

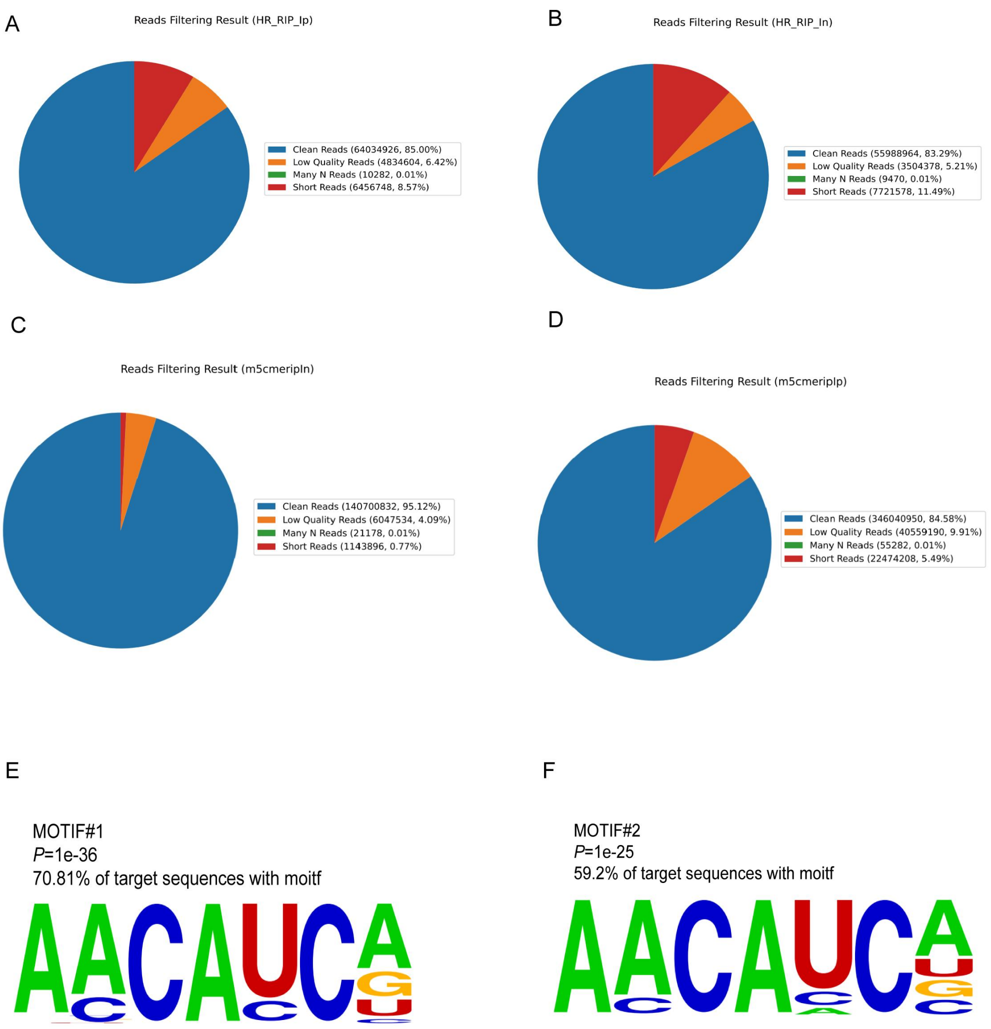

Fig. S4. (A-D) Sequencing data quality control analysis of Ripseq and m^{5}Cmeripseq. (E, F) Representative m^{5}C-associated sequence motifs from YBX1-bound transcripts.

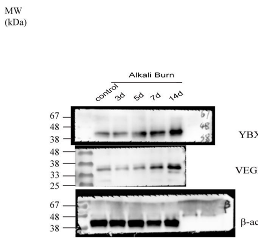

Fig. 2 D

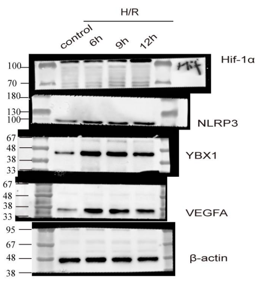

Fig. 3 J

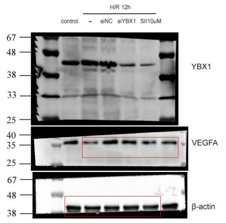

Fig. 4 B

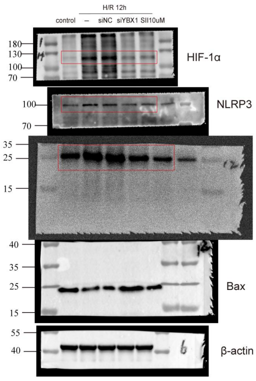

Fig. 5 A

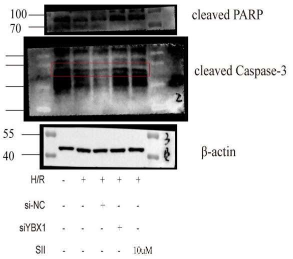

Fig. 5 F

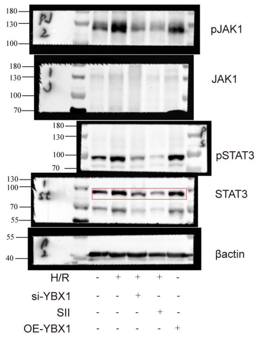

Fig. 5 I

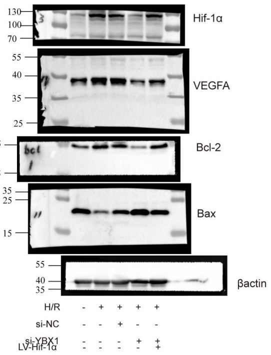

Fig. 6 A

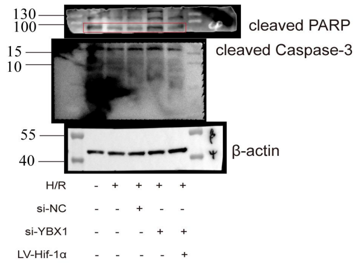

Fig. 6 F

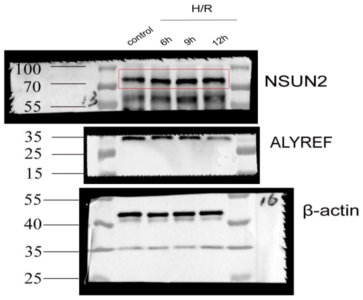

Fig. 7a A

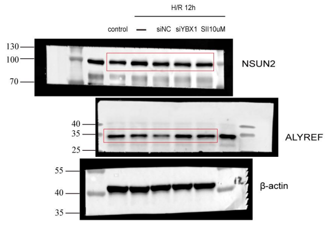

Fig. 7a D

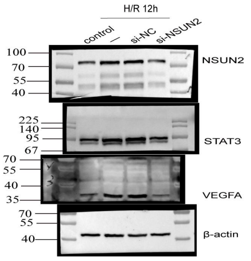

Fig. 7b A

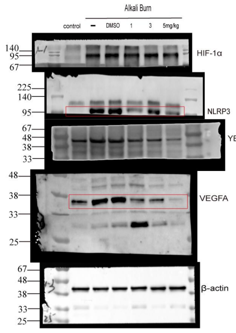

Fig. 8 G

Fig. S5 Uncropped images of wb results.
